# *Allium ursinum* L. as a Source of Natural Antioxidants: Phytochemical Diversity, Biological Effects, and Functional Food Applications

**DOI:** 10.3390/antiox15091144

**Published:** 2026-09-09

**Authors:** Cristina Adriana Rosan, George Alin Opris, Alexandra Cristina Tocai (Moțoc), Daniela Gitea, Ruben Budău, Manuel Alexandru Gitea, Simona Ioana Vicas

**Affiliations:** 1 Department of Food Engineering, Faculty of Environmental Protection, University of Oradea, 410048 Oradea, Romania; crosan@uoradea.ro (C.A.R.); opris.georgealin@student.uoradea.ro (G.A.O.); 2 Department of Preclinical Disciplines, Faculty of Medicine and Pharmacy, University of Oradea, 410087 Oradea, Romania; tocai.alexandra@gmail.com; 3 Department of Pharmacy, Faculty of Medicine and Pharmacy, University of Oradea, 410028 Oradea, Romania; dgitea@uoradea.ro; 4 Department of Silviculture and Forestry Engineering, Faculty of Environmental Protection, University of Oradea, 410048 Oradea, Romania; rbudau@uoradea.ro; 5 Department of Agriculture and Horticulture, Faculty of Environmental Protection, University of Oradea, 410048 Oradea, Romania; mgitea@uoradea.ro

**Keywords:** wild garlic, ramsons, organosulfur compounds, kaempferol derivatives, food authentication, bioaccessibility, nutraceuticals, clean-label ingredients, food processing, industrial applications

## Abstract

*Allium ursinum* L. (wild garlic) is a promising source of bioactive compounds for functional foods, nutraceuticals, and clean-label products. This review integrates current evidence on its phytochemical composition, biological activities, and food applications. The species contains sulfur compounds, polyphenols, vitamins, minerals, and pigments, whose levels vary with plant organ, developmental stage, environmental conditions, and processing. These constituents are associated mainly with antioxidant, antimicrobial, cardioprotective, and cytoprotective effects; however, evidence is derived predominantly from in vitro and animal studies, with limited clinical validation. In food systems, *A. ursinum* may enhance nutritional value, oxidative stability, sensory properties, and shelf life. Extraction, encapsulation, and stabilization technologies may support its use as a natural alternative to synthetic additives. Nevertheless, compositional variability, processing stability, bioavailability, authenticity, quality control, and raw-material supply remain major barriers to standardization and industrial application. Further research should combine phytochemical characterization with technological, safety, clinical, and sustainability assessments to enable reliable use of *A. ursinum* in food and nutraceutical products.

## 1. Introduction

In the context of increasing socioeconomic pressures and climate change, current food systems face growing challenges related to sustainability, human health, equity, and resilience [1]. This has stimulated growing interest in wild and underutilized plant species as resilient components of diversified and sustainable food systems [2]. Beyond their ecological relevance, these species are also being explored as potential sources of functional ingredients due to their richness in bioactive compounds capable of enhancing nutritional quality while supporting agricultural diversification [3].

Wild edible plants represent an important yet underexploited reservoir of phytochemicals, including polyphenols, flavonoids, carotenoids, vitamins, and minerals, which are associated with antioxidant and antimicrobial effects. However, despite increasing research interest, their integration into food systems remains limited by insufficient knowledge regarding phytochemical variability, lack of standardization, limited evidence on bioavailability, and the absence of clear links between chemical composition and functional performance in food matrices [3,4].

Within this context, the genus *Allium* includes numerous species of high nutritional and pharmacological relevance. A defining biochemical feature of this genus is the presence of sulfur-containing secondary metabolites, particularly S-alk(en)yl-L-cysteine sulfoxides (ACSOs), which act as precursors of volatile sulfur compounds formed upon tissue disruption. The enzymatic conversion of these precursors by alliinase leads to the formation of thiosulfinates, such as allicin, which are responsible for both the characteristic sensory properties and a significant part of the biological activity associated with *Allium* species [5,6,7].

*Allium ursinum* L., commonly known as wild garlic, ramson or ramsons, is a perennial geophyte belonging to the family Amaryllidaceae and is widely distributed throughout temperate regions of Europe [8]. The species is particularly associated with deciduous forest ecosystems, where it forms extensive populations [9,10], and has long been used as both a wild edible plant and a traditional medicinal resource [11,12].

Compared to cultivated species such as *Allium sativum*, *Allium ursinum* L. (wild garlic, ramsons) remains significantly less explored, despite exhibiting a comparable diversity of sulfur-containing compounds and a rich polyphenolic profile [13,14,15,16].

Nevertheless, comparative studies indicate that the two species are not phytochemically equivalent. Specifically, analyses of bulbs have described their organosulfur profiles as qualitatively similar but quantitatively different: *A. ursinum* contains relatively higher proportions of methiin (S-methyl-L-cysteine sulfoxide), methylallyl/allylmethyl thiosulfinate, methylajoene, and dimethylajoene, whereas *A. sativum* generally contains higher amounts of alliin and allicin [14,15,17]. Furthermore, the alliinase of *A. ursinum* differs from that of *A. sativum* in its substrate specificity and exhibits greater relative activity toward methiin [6]. Comparative in vitro studies also reported stronger inhibition of angiotensin-converting enzyme (ACE) by an aqueous *A. ursinum* leaf extract than by the corresponding *A. sativum* leaf extract under the tested conditions [14]. These phytochemical, enzymatic, and functional particularities support the consideration of *A. ursinum* as a distinct source of bioactive compounds rather than merely a substitute for cultivated garlic.

In recent years, interest in *A. ursinum* has increased due to its dual role as a traditional medicinal plant and a potential functional food ingredient, with reported antimicrobial and cardioprotective effects [18,19,20].

However, current knowledge remains fragmented, as studies generally focus on phytochemical composition, biological activity or technological applications separately, with limited integration across these domains [13,16,21,22]. At the same time, most reported biological activities are based on in vitro or animal studies, with limited clinical validation [19,20]. More specifically, no controlled human efficacy trials were identified, while the available human evidence is limited to a small metabolic study and to ex vivo or in vitro investigations using human-derived cells [23,24,25,26]. From a food science perspective, critical aspects such as processing stability, bioactive compound retention, and industrial scalability remain insufficiently addressed [27,28]. Food-matrix studies predominantly address technological, physicochemical, antioxidant, antimicrobial, and sensory outcomes, while evidence on bioaccessibility is limited to simulated gastrointestinal digestion and human bioavailability or pharmacokinetic data are currently unavailable [29]. Additionally, the risk of misidentification with morphologically similar toxic species such as *Convallaria majalis* and *Colchicum autumnale* represents a safety concern, particularly in the context of wild harvesting [30,31,32].

Given these limitations, a comprehensive evaluation of *A. ursinum* is needed. Therefore, this review aims to synthesize and critically discuss the current knowledge regarding its botanical characteristics, phytochemical composition, biological activities, and food-related applications, with particular emphasis on identifying key limitations, methodological inconsistencies, and research gaps. The novelty of this review lies in its integrated assessment of phytochemical composition, biological evidence, processing stability, and functional performance in food matrices, which are frequently considered as separate domains in the available literature. Furthermore, the review explores the relationship between chemical composition and functional properties and highlights future research directions required for the effective integration of *A. ursinum* into functional food and nutraceutical applications.

## 2. Methodology

### 2.1. Literature Search and Study Selection

A literature search was conducted in the Web of Science Core Collection and PubMed databases to identify studies related to *Allium ursinum* L. The literature search was updated and finalized on 22 August 2026. The search strategy was designed to maximize sensitivity while accounting for the taxonomic ambiguity of vernacular names used for the species.

In the Web of Science Core Collection, the primary search was performed in the Topic field using the exact query TS = (“*Allium ursinum*”), which retrieved 253 records. To identify potentially relevant publications that did not contain the scientific name, an additional search was performed using the vernacular terms “wild garlic” and “ramson”. The query TS = ((“wild garlic” OR ramson) NOT “*Allium ursinum*”) retrieved 112 additional records.

Because “wild garlic” and “ramson” are not taxonomically specific and may refer to other *Allium* species or occur in unrelated contexts, all 112 additional records were individually screened for taxonomic relevance. Initial screening identified 9 records explicitly referring to *A. ursinum*, 5 records considered probably related to the species, 5 records with uncertain taxonomic identity, and 93 records referring to other species or representing false-positive or irrelevant results. The 10 probable or uncertain records were subsequently subjected to detailed manual verification. Eight were confirmed as referring to *A. ursinum,* and two were excluded. Consequently, 17 of the 112 additional records were retained, and 95 were excluded as taxonomically irrelevant or false-positive results. Together with the 253 records retrieved using the scientific name, this resulted in 270 taxonomically relevant Web of Science records.

In PubMed, the final search was performed using the query (“*Allium ursinum*”[Title/Abstract]) OR (ramson[Title/Abstract]), which retrieved 109 records. No language restriction was applied during database searching.

All references from both databases were exported and imported into Zotero (version 10.0.1) reference management software. Cross-database deduplication was subsequently verified using PMID, DOI, and normalized publication titles. Of the 253 records retrieved through the scientific-name search in Web of Science and the 109 PubMed records, 96 duplicate records were identified, resulting in 266 unique records. Of the 17 additional Web of Science records confirmed through the vernacular-name search, one was already represented in the PubMed dataset; therefore, 16 additional unique records were added. The combined dataset consequently comprised 282 unique records before eligibility screening.

Titles and abstracts were screened for relevance to the objectives of the review. Studies were excluded when *A. ursinum* was mentioned only incidentally, when the research focused on other *Allium* species without providing specific data for *A. ursinum*, when publications were unrelated to the scope of the review (e.g., zoological studies, unrelated ecological surveys, humanities, or social sciences). At this stage, 143 records were excluded, leaving 139 publications for detailed eligibility assessment.

Full-text availability was not used as an independent exclusion criterion. When the full text could not be readily accessed, bibliographic records, abstracts, and other available publication information were examined to determine eligibility whenever sufficient information was available. During the detailed eligibility assessment, 60 additional publications were excluded because they provided insufficient extractable species-specific information, addressed peripheral topics outside the predefined synthesis domains, represented broader comparative studies without sufficiently separable data for *A. ursinum*, or provided insufficient methodological detail for reliable evidence synthesis. Consequently, 79 publications were retained for the final qualitative synthesis.

Eligible studies addressed one or more of the following aspects: phytochemical composition, sulfur-containing compounds, polyphenols and antioxidants, nutritional composition, biological activities (antioxidant, antimicrobial, anti-inflammatory, etc.), food applications and functional food potential, and processing or technological aspects.

Original research articles were eligible when they provided species-specific information relevant to these topics. Review articles were used to contextualize the evidence and to identify relevant primary literature but were distinguished from primary experimental evidence during synthesis. Full conference/proceedings papers were considered eligible when they contained sufficiently detailed methodological and species-specific information, whereas meeting abstracts lacking sufficient methodological information for evidence assessment were excluded. Book chapters and dissertations/theses were eligible in principle when they fulfilled the same relevance and information requirements; however, no such document types were identified among the 282 unique database records subjected to screening. Publications were not excluded solely on the basis of language when sufficient information was available for reliable interpretation.

### 2.2. Data Extraction, Evidence Assessment, and Synthesis

For each eligible publication, information regarding publication year, plant material, analytical methods, identified bioactive compounds, biological activities, and food-related applications was extracted and synthesized.

Because of the substantial methodological heterogeneity among the included studies, including differences in plant material, extraction procedures, analytical methods, experimental models, concentrations, and reported outcomes, a quantitative meta-analysis was not considered appropriate. The extracted evidence was therefore synthesized qualitatively and organized according to the principal topics addressed in the review.

During evidence synthesis, studies were critically evaluated according to the type and level of evidence provided. For experimental studies, particular attention was given, where reported, to plant material and its identification, sample size or number of replicates, experimental model, analytical methodology, presence of appropriate controls, treatment or extract concentration, and consistency between the reported results and conclusions. For studies reporting biological activities, evidence derived from chemical or cell-free assays, cellular or other in vitro models, animal studies, food-matrix studies, and human studies was distinguished to avoid interpreting findings obtained at different experimental levels as equivalent evidence.

Methodological limitations and comparability between studies were also considered when interpreting reported effects. Particular caution was applied when studies differed substantially in extraction solvent, plant organ, phenological stage, geographical origin, analytical procedure, or expression of results. Consequently, biological activities observed under specific experimental conditions were interpreted as evidence of potential activity rather than as direct evidence of clinical efficacy or health benefit.

### 2.3. Bibliometric Analysis

Bibliometric analysis was conducted separately from the literature search and qualitative evidence synthesis described in Section 2.1 and Section 2.2. Its purpose was to characterize publication trends and the thematic structure of *A. ursinum* research rather than to determine eligibility for the qualitative review.

A separate bibliometric dataset comprising 270 taxonomically validated records was derived exclusively from the Web of Science Core Collection using the search strategy described in Section 2.1. Web of Science was selected because its standardized bibliographic metadata are suitable for bibliometric mapping and keyword co-occurrence analysis. In contrast, the literature review combined Web of Science and PubMed records and comprised 282 unique records before eligibility screening. Duplicate, taxonomically irrelevant, and false-positive records were removed before bibliometric analysis. Self-citations were not removed because the analysis was based on keyword co-occurrence rather than citation relationships.

The records were exported as Full Record and Cited References in plain text format and analyzed using VOSviewer (version 1.6.21).

Keyword co-occurrence analysis was performed using Author Keywords and Keywords Plus with a minimum occurrence threshold of four. From 1647 identified keywords, 95 met the threshold. To improve network readability and thematic interpretability, the 60 most relevant keywords were retained for visualization.

**Figure 1:**
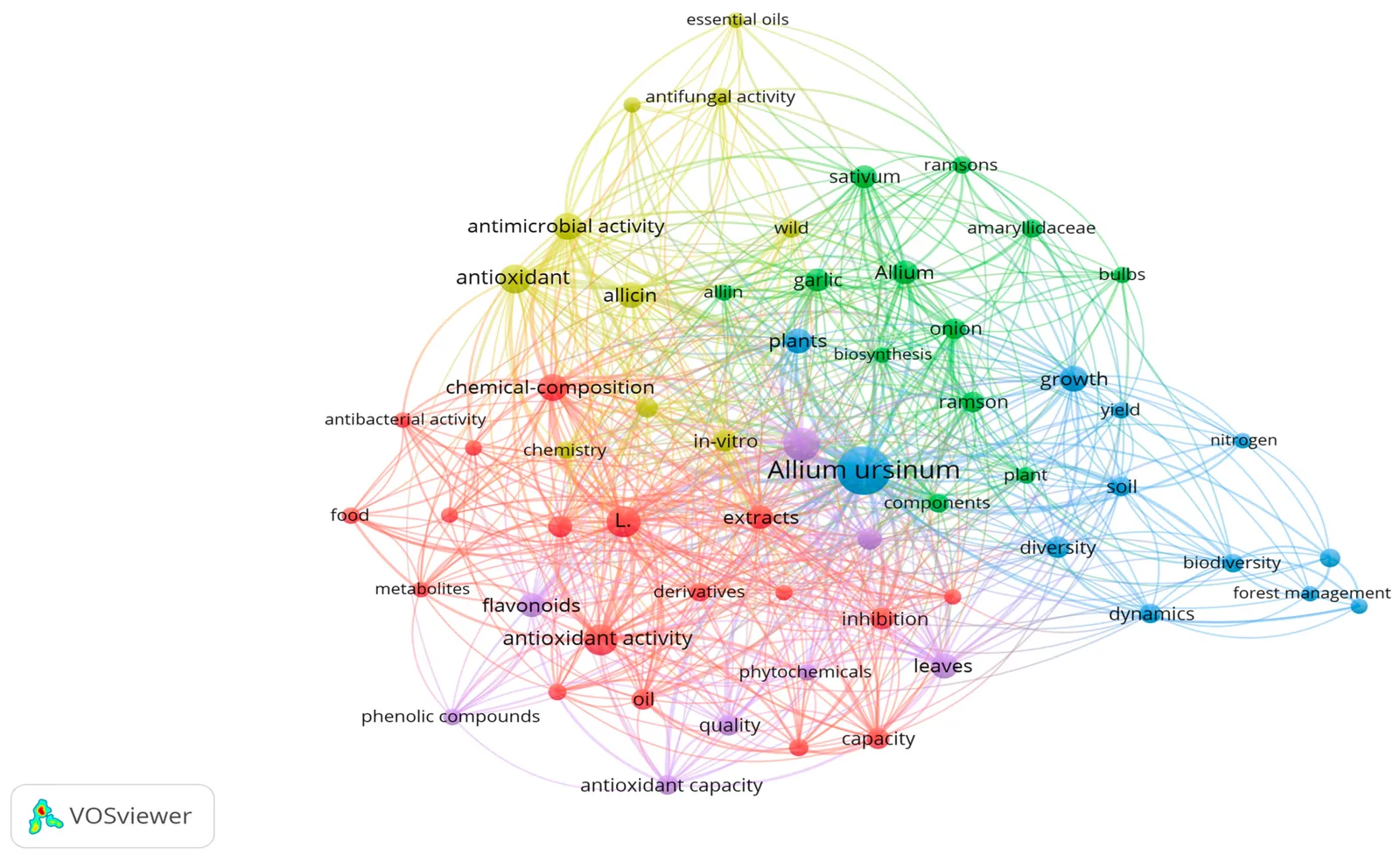
Keyword co-occurrence network of *Allium ursinum* L. research based on 270 taxonomically validated Web of Science Core Collection records and visualized using VOSviewer (version 1.6.21; minimum keyword occurrence threshold = 4). Of 1647 identified keywords, 95 met the threshold, and the 60 most relevant terms were retained for visualization. Node size reflects keyword occurrence frequency, link thickness indicates co-occurrence strength, and colors represent the five thematic clusters.

Before network construction, a thesaurus file was applied to standardize graphical variants of the scientific name *Allium ursinum* (including “*Allium ursinum*”, “*Allium-ursinum*”, and “*Allium ursinum* L.”). Vernacular terms such as “wild garlic”, “ramson”, and “ramsons” were deliberately retained as separate terms because these common names are not taxonomically specific (Figure 1).

The keyword co-occurrence analysis identified five interconnected clusters representing the major research directions in the *A. ursinum* literature. These were broadly associated with phytochemical composition and biological activity, the botanical and biochemical context of the genus *Allium*, ecological and environmental research, sulfur chemistry and antimicrobial activity, and bioactive compounds and quality-related characteristics. The *Allium ursinum* node occupied a central position in the network, indicating strong connections across these research themes.

Overall, the bibliometric analysis indicates that research on *A. ursinum* is predominantly focused on phytochemical characterization and the evaluation of biological activities, particularly antioxidant and antimicrobial properties.

## 3. Botanical, Taxonomic and Ecological Characteristics of *Allium ursinum* L.

A comprehensive understanding of *Allium ursinum* L. requires an integrated analysis of its taxonomic classification, morphological characteristics, geographic distribution, and cytogenetic features. These aspects provide essential information for accurate species identification and form the basis for evaluating its biological properties and potential applications.

### 3.1. Taxonomic Classification and Nomenclature

The genus *Allium* comprises more than 1000 species and represents one of the largest genera within the family Amaryllidaceae [33,34]. Contemporary classification places *Allium ursinum* L. within subgenus *Amerallium* Traub and section *Arctoprasum* Kirschl. [16,35]. The species name *ursinum* originates from the Latin word *ursus* (“bear”), reflecting traditional beliefs associated with the plant. Several synonyms have been reported, including *Allium nemorale* Salisb., *Allium latifolium* Gilib., and *Ophioscorodon ursinum* (L.) Wallr. [16]. According to the Catalogue of Life [8], the currently accepted taxon is *Allium ursinum* subsp. *ursinum*. The current taxonomic classification of the species is presented in Table 1.

### 3.2. Morphological Description

*Allium ursinum* L. is a perennial bulbous, vernal geophyte adapted to the seasonal dynamics of deciduous forest understories. The species usually forms dense populations and may reach up to 50 cm in height under favorable conditions [10].

**Figure 2:**
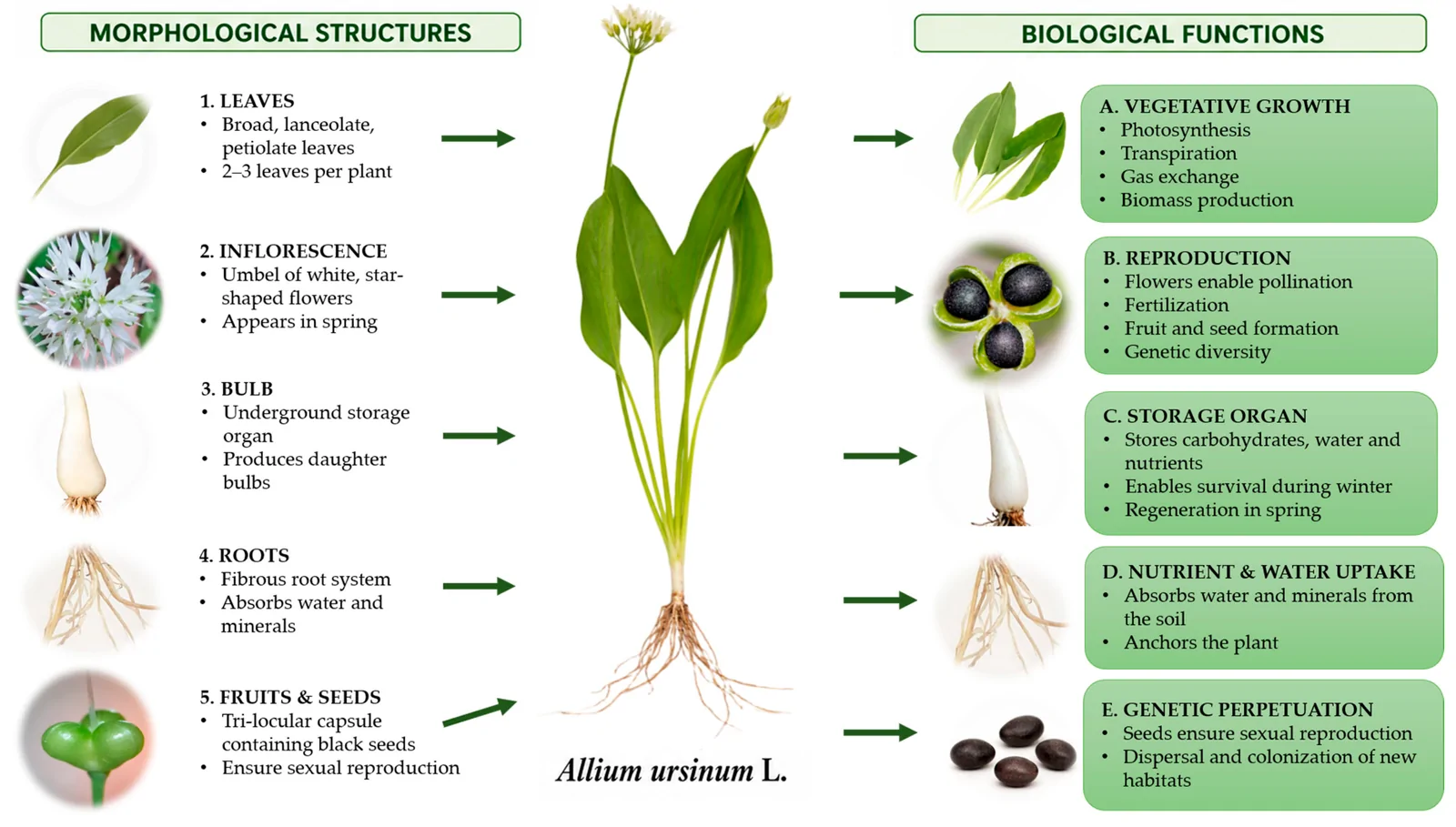
Morphological organs and associated biological functions of *Allium ursinum* L. Arrows indicate the relationships between the morphological structures and their associated biological functions.

The plant develops a narrow, elongated bulb, approximately 1.5–6 cm long, surrounded by translucent tunics. The aerial part consists of an erect, triquetrous flowering stem and usually 2–3 smooth, elliptic-lanceolate to lanceolate leaves, which are shorter than the stem and gradually narrow into a petiole. The inflorescence is a terminal, umbel-like structure composed of star-shaped white flowers; literature data indicate broad variation in flower number, ranging from 3 to 30 flowers per inflorescence, with cultivated plants frequently producing about 13–24 flowers [10,36]. The species reproduces mainly by seeds, although vegetative propagation through daughter bulbs may also occur. Seeds are black and subglobose, enclosed in capsules and released after flowering. The main morphological organs and their functional roles are illustrated in Figure 2.

From an infraspecific perspective, the two recognized subspecies also show diagnostic morphological differences, particularly in pedicel surface. *A. ursinum* subsp. *ursinum* has rough, papillate pedicels, whereas *A. ursinum* subsp. *ucrainicum* is characterized by smooth pedicels without papillae [10,35,37].

Beyond its general morphology, *A. ursinum* exhibits considerable phenotypic variability. Traits such as leaf size, petiole length, flowering parameters, bulb development and biomass production vary among populations and are influenced by habitat conditions, altitude, soil properties and local climatic factors [9,10,36].

### 3.3. Geographic Distribution and Ecological Requirements

**Figure 3:**
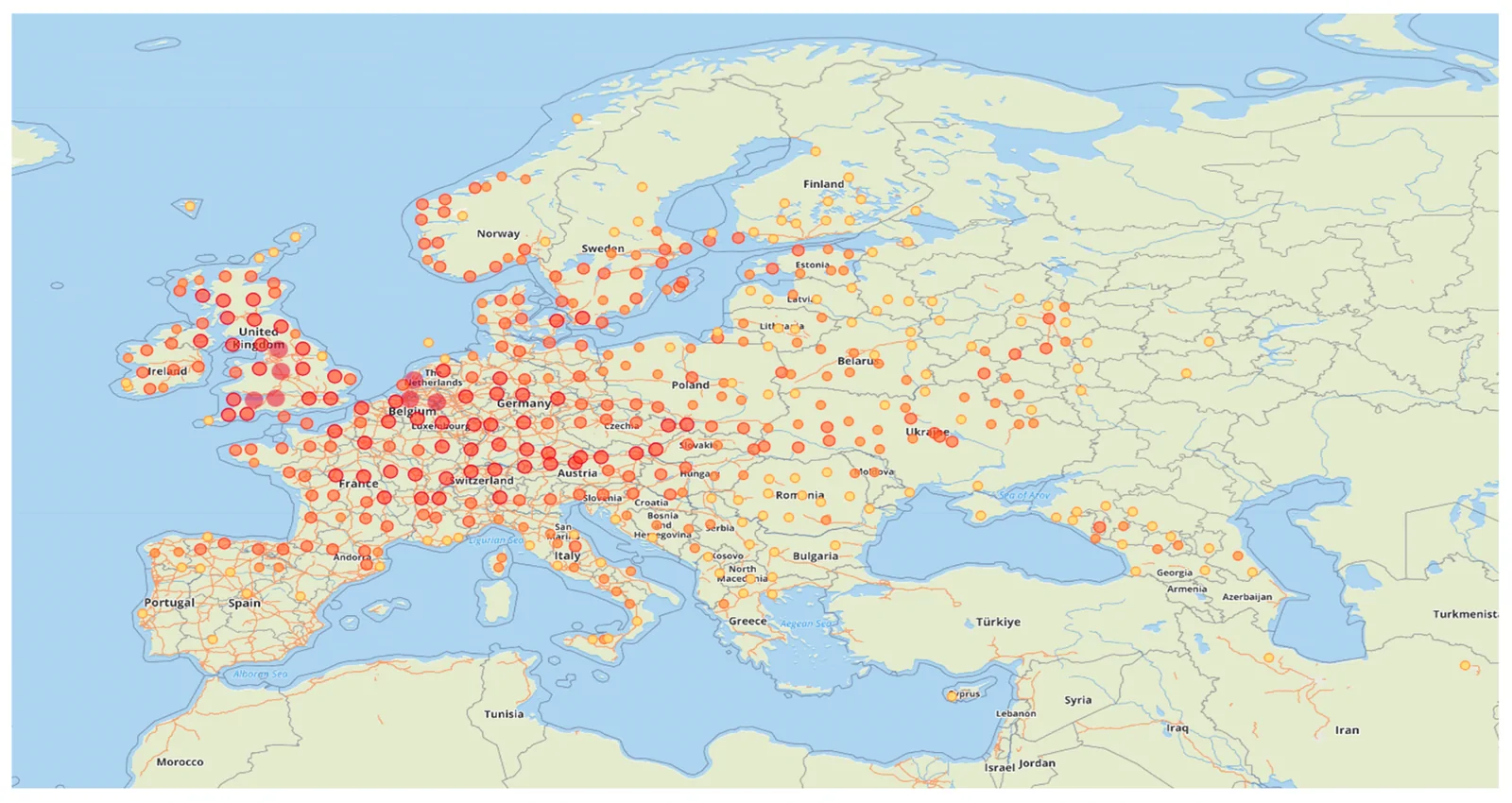
Geographic distribution of *Allium ursinum* L. in Europe based on occurrence records obtained from the Global Biodiversity Information Facility (GBIF). Circle size reflects the number of occurrence records aggregated at the displayed map scale; symbol color is part of the GBIF map visualization and does not represent a separate biological category. The records represent reported georeferenced occurrences and should not be interpreted as estimates of species abundance, population density, or population size. The map was generated using GBIF occurrence data and OpenStreetMap base-map layers. GBIF Occurrence Download. Available online: https://doi.org/10.15468/dL.cxww5w (accessed on 31 July 2026). Map data © OpenStreetMap contributors [38].

*Allium ursinum* L. is a Eurasian species widely distributed throughout temperate regions of Europe and parts of western Asia. According to records from the Global Biodiversity Information Facility (GBIF), the species is represented by more than 200,000 georeferenced occurrences, with the highest concentration located in Central and Western Europe (Figure 3). These records represent reported georeferenced observations and should not be interpreted as direct estimates of species abundance, population density, or population size. Its distribution extends from the Iberian Peninsula and Western Europe to Eastern Europe, the Balkans, the Caucasus, and Anatolia [10,35].

The species is typically associated with moist, shaded deciduous forests, particularly communities dominated by *Fagus sylvatica*, *Carpinus betulus*, and other broadleaf species [10,39]. As a spring geophyte, *A. ursinum* completes most of its vegetative growth before canopy closure, often forming dense populations in forest understories [39]. This seasonal growth strategy makes light availability an important component of its ecological niche, as much of the above-ground development occurs during the relatively high-light period preceding full canopy closure.

Its occurrence is strongly influenced by soil conditions. *A. ursinum* prefers humus-rich, nutrient-rich, well-drained soils with neutral to slightly acidic pH and adequate moisture, whereas excessively dry, sandy, or aluminum-rich soils restrict its distribution [16,40]. Beyond their effects on species occurrence and growth, environmental conditions may also contribute to the considerable phytochemical variability observed among wild populations. Studies conducted across natural habitats have shown that differences in soil properties and nutrient status are associated with variation in phenolic and flavonoid contents, with soil characteristics representing an important source of variability in bioactive compound concentrations [9]. Climatic conditions may exert an additional influence: comparative analysis of natural populations showed significant differences in phytochemical profiles and antioxidant potential, with higher precipitation and moderate spring temperatures being associated with higher phenolic contents [41].

Phenological stage represents another important source of chemical variability. The qualitative and quantitative profiles of sulfur-containing compounds change during the vegetation period, with higher amounts of volatile precursors reported in March and April, shortly before flowering [6]. Likewise, phenolic contents differ between developmental stages [42], while maturity has also been shown to influence carotenoid, chlorophyll and triterpenoid contents as well as antioxidant capacity [43]. These findings indicate that differences in harvest period and phenological stage should be considered when comparing the phytochemical composition of wild *A. ursinum* populations.

Light conditions, nutrient availability, and habitat disturbance may additionally influence plant performance and population dynamics. However, their direct effects on the phytochemical composition of *A. ursinum* are less comprehensively documented than those of phenological stage, soil characteristics, and climatic conditions; therefore, these ecological variables should be regarded as potential contributors to the variability observed among wild populations [44].

Because of its ecological association with moist, nutrient-rich deciduous forests, *A. ursinum* has been used as an indicator species of good-quality forest stands in ecological assessments [45].

The species exhibits considerable ecological plasticity and can occur from lowland habitats to montane forests, depending on regional environmental conditions [9,10].

### 3.4. Cytogenetic Features and Genome Size

The genus *Allium* is characterized by considerable cytological and genomic diversity [46]. Although the most common basic chromosome number in the genus is x = 8, other numbers, including x = 7, 9, 10, and 11, as well as variation in ploidy, have also been reported [46]. In this context, *A. ursinum* represents a species with the basic chromosome number x = 7.

A notable feature of *A. ursinum* is its very large nuclear genome [47]. Its genome size has been estimated at approximately 1C = 31.45 pg (≈30.76 Gb), which is almost twice the genome size reported for cultivated relatives such as *A. cepa* and *A. sativum* [47,48]. This places *A. ursinum* among the large-genome representatives of the genus *Allium*.

Available genomic studies also indicate that *A. ursinum* differs from cultivated *Allium* species in its repetitive DNA composition. Comparative repeatome analyses of *A. cepa*, *A. sativum*, and *A. ursinum* revealed species-specific differences in tandem repeats and other repetitive elements, supporting the genomic distinctiveness of *A. ursinum* within the genus [48].

At the plastome level, *A. ursinum* appears relatively conserved compared with some other members of subgenus *Amerallium*. Complete plastome sequencing showed that the *A. ursinum* plastome contains 90 predicted genes, including 81 unique genes and five pseudogenes, and lacks the major rearrangements observed in *A. paradoxum* [49].

Population-level molecular studies have further revealed genetic differentiation among *A. ursinum* populations and subspecies, particularly across the contact zone between *A. ursinum* subsp. *ursinum* and *A. ursinum* subsp. *ucrainicum*, indicating the existence of geographically structured genetic variation within the species [50,51].

The botanical characteristics, ecological adaptation, and widespread distribution of *A. ursinum* have contributed to its long-standing use as both a food and medicinal plant throughout Europe.

Taken together, genetic background, geographical origin [50], habitat conditions, and developmental stage provide an important framework for interpreting the compositional variability of *A. ursinum* [51]. These factors should therefore be considered when comparing the nutritional and phytochemical data discussed in the following sections.

## 4. Traditional Uses and Contemporary Culinary Applications of *Allium ursinum* L.

### 4.1. Ethnobotanical and Traditional Uses

*Allium ursinum* L. (wild garlic, ramsons, or bear’s garlic) has a long history of use as both a food and medicinal plant throughout Europe. Its early spring emergence made it an important seasonal food resource, particularly in rural and mountainous regions, where wild edible plants traditionally supplemented local diets after winter [11,16]. The leaves were commonly consumed raw, used as a seasoning, or cooked as a vegetable.

Archaeobotanical evidence suggests that *A. ursinum* was consumed as early as the Mesolithic period. Charred bulbs recovered from the late Mesolithic settlement of Halsskov in Denmark indicate that the species may have formed part of the hunter-gatherer diet [52]. Archaeobotanical records from medieval West Slavic settlements provide additional evidence of the historical presence and possible cultivation of *A. ursinum* [53]. The species was also described in historical European herbals [54,55].

Besides its culinary importance, *A. ursinum* has been widely used in European folk medicine, with some applications resembling those attributed to cultivated garlic, *Allium sativum* L. Traditional applications included its use as a digestive stimulant, a perceived detoxifying and antimicrobial remedy, and for respiratory, cardiovascular, skin and wound-related complaints [12,55,56].

More specifically, Irish ethnomedicinal records describe the use of the leaves for chest and lung infections, coughs, colds, asthma, and bronchitis, as well as for hypercholesterolemia and reducing blood clot formation [56]. Among Romanian communities in Bukovina, the leaves were traditionally used for perceived detoxification and immune support [11], whereas a recent ethnobotanical survey from the central Balkans documented the use of *A. ursinum* for circulatory system disorders [57].

These ethnobotanical records document historically and culturally transmitted practices and should not be interpreted as evidence of clinical efficacy. Nevertheless, they provide an important context for understanding the long-standing use of *A. ursinum* and the subsequent scientific investigation of its phytochemical composition and biological properties.

### 4.2. Culinary Applications

In contemporary culinary practice, *Allium ursinum* L. is used mainly as a flavoring ingredient due to its characteristic garlic-like aroma, which is largely associated with sulfur-containing compounds and their degradation products [12,16]. Its leaves are incorporated into both raw and cooked dishes, where they provide a fresh, herbaceous and mildly pungent sensory profile.

Recent studies show that the culinary use of *A. ursinum* has expanded beyond household preparations toward formulated food products. In pasta, wild garlic has been used as powder, extract, encapsulated extract, fresh shredded leaves, or blanched chopped leaves. These forms improve the phytochemical profile and antioxidant capacity of pasta while maintaining acceptable sensory quality, especially at low or moderate incorporation levels [27,58,59].

Wild garlic is also increasingly used in dairy-based foods. The addition of its leaves to soft rennet-curd cheeses modifies the volatile profile, reduces undesirable milky, sour, or sheep-milk notes, and enhances herbal or piquant attributes without negatively affecting overall sensory quality [28,60]. In fermented sheep milk kefir, 1% freeze-dried wild garlic powder acted as a taste and flavor modifier, although consumer preference may depend on familiarity with the garlic-like note [61].

Beyond its technological role, *A. ursinum* has become a marker of regional gastronomy. In rural Serbian destinations, it is associated with seasonal harvesting, local dishes, pesto, pickled products, gastronomic events, and culinary heritage, supporting its role as a bridge between traditional cuisine and contemporary gastronomic innovation [62].

Beyond these culinary uses, *A. ursinum* has been incorporated into a broader range of food matrices, including meat products, mayonnaise, extruded cereal products, and other functional food formulations. The effects of fresh, blanched, dried, and otherwise processed forms on phytochemical retention, antioxidant activity, technological properties, color, aroma, and sensory quality depend strongly on the processing conditions applied. Processing-induced changes in sulfur compounds and other phytochemicals are examined in Section 6, whereas their implications for technological performance and sensory quality in specific food matrices are discussed in Section 8. Given the frequent use of wild-harvested material, safety considerations related to correct botanical identification, toxic lookalike species, authentication, and quality control of commercial raw materials are addressed separately in Section 7.6.

## 5. Nutritional Composition of *Allium ursinum* L.

*Allium ursinum* L. is increasingly regarded as a nutritionally relevant wild edible species because its leaves, flowers and bulbs contain primary nutrients, vitamin C, minerals, dietary fiber and other nutritionally relevant constituents. Published nutritional values vary according to biological material and reporting basis; comparisons should therefore consider plant organ, sampling conditions, and whether results are expressed on a fresh- or dry-weight basis [43,63,64,65].

For the purpose of this review, nutritional constituents are distinguished from specialized bioactive phytochemicals. Accordingly, Section 5 focuses on proximate composition, vitamin C, minerals and other nutritionally relevant constituents, whereas sulfur-containing compounds, polyphenols, carotenoids, pigments and other specialized metabolites are discussed in detail in Section 6. Carotenoids are considered in Section 5.4 only in relation to their nutritional relevance, together with the available evidence on other constituents, such as organic acids; their detailed phytochemical characteristics are addressed in Section 6.3 and Section 6.4, respectively.

**Figure 4:**
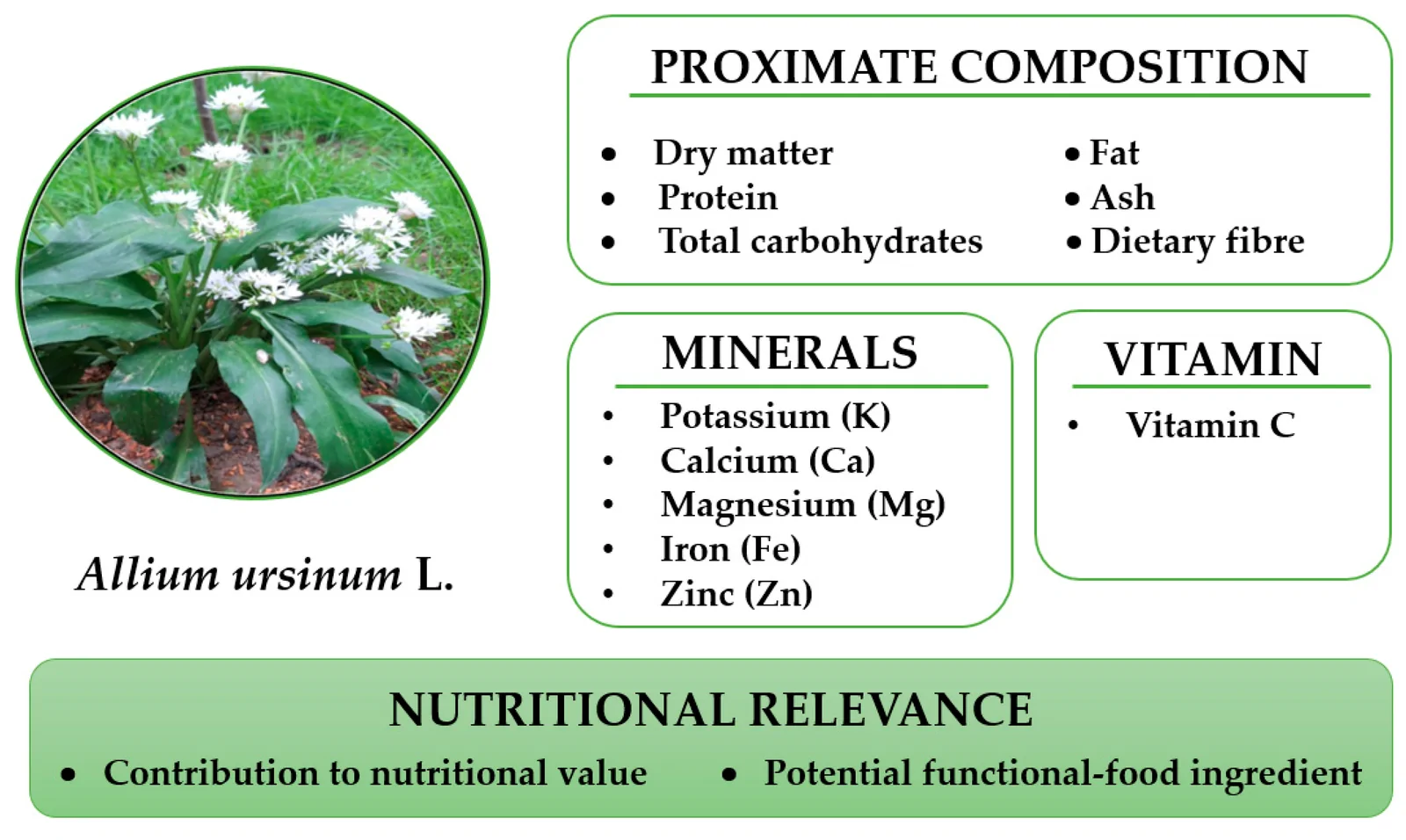
Main nutritional components contributing to the nutritional value of *Allium ursinum* L.

To facilitate the interpretation of the nutritional profile of *A. ursinum*, Figure 4 summarizes the principal nutritional components reported for its edible tissues. These include proximate constituents, dietary fiber, vitamin C, minerals and other nutritionally relevant compounds, while specialized bioactive phytochemicals are addressed separately in Section 6.

### 5.1. Proximate Composition

In this review, proximate composition is used in its conventional analytical sense and includes dry matter, protein, fat, carbohydrates, dietary fiber, and ash. Dry matter represents the non-water fraction of the plant material, whereas ash represents the inorganic residue remaining after incineration.

The proximate composition of *A. ursinum* L. differs markedly among plant organs. Bulbs show higher dry-matter contents than leaves and stems, whereas leaves contain higher protein and lipid concentrations when compared on a dry-matter basis [63]. This also explains why values expressed on a fresh mass basis appear lower than dry matter-based values reported for the same or other organ. In addition, part of the apparent disagreement between studies results from differences in reporting basis and analytical methodology, as well as from the use of different approaches for carbohydrate determination [63,65]. Consequently, fresh-weight data should not be directly compared with dry-matter data without explicit conversion or careful contextualization [64,65]. The available data on the proximate composition of different plant organs of *A. ursinum* are summarized in Table 2.

The data summarized in Table 2 indicate that the proximate composition of *Allium ursinum* L. varies among plant organs and studies. Dry matter content was higher in bulbs (26.74–27.79 g/100 g FW), than in leaves and stems (approximately 9–11 g/100 g FW), although variation in developmental stage, sampling conditions, and analytical procedures should also be considered. Organ-dependent differences were also reported for protein, lipid, and ash contents [63].

When expressed on a dry-weight basis, the nutritional composition of *A. ursinum* varies markedly among plant organs. Jędrszczyk et al. (2019) reported leaf protein concentrations of 19.33–26.53 g 100 g^−1^ DW, compared with 6.23–16.12 g 100 g^−1^ DW in stems [63]. Across the two experimental years, the mean dry matter content was approximately 10.28% in leaves, 10.43% in stems and 27.26% in bulbs. Leaves had the highest mean protein (22.93 g 100 g^−1^ DW), lipid (3.65 g 100 g^−1^ DW) and ash contents (7.73 g 100 g^−1^ DW), whereas stems contained the highest mean total carbohydrate concentration (4.58 g 100 g^−1^ DW). Analyses conducted on a fresh-weight basis also revealed considerable variation among studies. Piątkowska et al. reported 13.7 g kg^−1^ FW protein and 50.8 g kg^−1^ FW total carbohydrates in fresh leaves, whereas Luță et al. found 4.03% FW protein and 1.34 ± 0.12% FW total soluble sugars. More recently, Ļubina et al. (2025) reported 4.0 g protein, 0.6 g fat, and 2.5 g dietary fiber per 100 g of frozen wild garlic [68]. Because these values were reported per 100 g of the analyzed material without an explicit FW or DW designation, they are retained in Table 2 on the original reporting basis and should not be directly compared with values expressed specifically on a dry-weight basis. However, these values are not directly comparable because total carbohydrates encompass a broader range of compounds, whereas the Somogyi-Nelson method used by Luță et al. quantified the soluble sugar fraction. Additional variation may reflect differences in sampling and analytical methodology. Particular attention should be paid when comparing carbohydrate values among studies.

Piątkowska et al. reported total carbohydrates as 50.8 g kg^−1^ FW, whereas Jędrszczyk et al. reported lower values expressed on a dry matter basis. Such differences are unlikely to reflect biological variability alone and may also result from methodological differences, including the calculation of carbohydrates by difference versus the determination of specific carbohydrate fractions such as soluble sugars. Although the available literature confirms the presence of a nutritionally relevant carbohydrate fraction in *A. ursinum* leaves, quantitative information on individual sugars remains considerably more limited than data on total carbohydrates or total soluble sugars. Therefore, total carbohydrate and soluble-sugar values are reported separately in Table 2 and should not be interpreted as analytically equivalent parameters. Similar considerations apply to fat and ash contents, whose apparent variability is amplified by the use of fresh-weight and dry-matter reporting systems.

In addition to the carbohydrate fractions reported in previous studies, Čepulienė et al. determined a total soluble solids content of 12.0 ± 0.29% in fresh *A. ursinum* leaves. Although total soluble solids do not represent a specific nutrient class, this parameter provides complementary information on the soluble constituents of fresh leaf tissue [67].

Seasonal variability may also contribute to differences in nutrient accumulation, as illustrated by the two-year data reported by Jędrszczyk et al. [63]. Accordingly, proximate composition should be interpreted as variable rather than fixed.

### 5.2. Vitamin C Content and Variability

Vitamin C content in *A. ursinum* L. is variable and is particularly sensitive to harvest stage, environmental exposure, and postharvest handling [63]. Consequently, differences among studies are expected and should be interpreted within the context of sample origin and analytical procedure. The available literature data regarding vitamin C concentrations in different organs of *A. ursinum* are summarized in Table 3.

Vitamin C concentrations showed a clear organ-dependent distribution pattern, with the highest contents recorded in leaves (24.20–49.70 mg 100 g^−1^ FW), intermediate levels in stems (18.47–41.14 mg 100 g^−1^ FW), and the lowest concentrations in bulbs (5.43–6.11 mg 100 g^−1^ FW) [63]. These results demonstrate an organ-dependent distribution of vitamin C, with substantially higher concentrations in the aerial tissues than in bulbs; however, the available compositional data do not allow this pattern to be directly attributed to specific physiological functions.

Fresh leaves collected from Romanian wild populations contained 79.84 ± 2.76 mg 100 g^−1^ FW vitamin C [66], whereas six Latvian accessions analyzed by HPLC-DAD contained 16.5–37.8 mg 100 g^−1^ FW [13]. Similarly, Croatian populations evaluated using spectrophotometric methods showed concentrations ranging from 23.4 to 49.8 mg 100 g^−1^ FW depending on location and phenological stage [70]. These differences likely reflect both biological and methodological variability among studies. Despite this variability, all studies consistently identify leaves as the richest source of vitamin C within the plant. The concentrations of ascorbic acid reported for fresh leaves indicate that vitamin C contributes to the nutritional composition of *A. ursinum* and may also contribute to its antioxidant potential in food applications [13,63,66,70].

### 5.3. Mineral Composition

Mineral composition in *Allium ursinum* L. varies considerably among habitats and may be influenced by environmental and edaphic conditions, geographical origin, and analytical methodology [71]. Consequently, differences among published datasets should not be interpreted as contradictory but rath.șmer as reflecting environmental variability and methodological diversity. Furthermore, mineral concentrations are reported either on a fresh-weight (FW) or dry-matter (DW) basis, making direct comparisons among studies difficult without considering the expression basis. The quantitative mineral data available for *A. ursinum* leaves are summarized in Table 4.

The mineral profile of *A. ursinum* indicates appreciable concentrations of several macro- and microelements, particularly potassium, calcium, magnesium, and iron. Regardless of the analytical methodology or reporting basis, potassium was consistently identified as the predominant macronutrient, followed by calcium and magnesium, whereas iron and zinc were among the nutritionally relevant trace elements [63,64,65,71].

At first glance, the mineral concentrations summarized in Table 4 appear highly variable. However, these differences are largely explained by three major factors: the basis of expression (fresh weight versus dry matter), environmental variability, and analytical methodology. The use of different reporting bases can have a pronounced effect on apparent mineral concentrations. Consequently, direct comparisons among studies require careful consideration of the expression basis and units used. Consequently, comparisons between studies should only be made after considering the moisture basis used [64,65].

Environmental conditions constitute another important source of variability. Gordanić et al. reported substantial variation in leaf elemental composition across 43 locations in Serbia and related these differences to geographical and agroecological variability [71].

Methodological differences further complicate direct comparisons. The studies summarized in Table 4 employed different analytical techniques, including AAS, ICP-AES, ICP-OES, and spectrophotometric determination [63,64,65,71]. Although these techniques are well established for elemental analysis, differences in sample digestion, calibration procedures, instrumental sensitivity, and reporting units may contribute to quantitative discrepancies, particularly for trace elements such as copper, zinc, and manganese.

From a nutritional perspective, however, total mineral concentration alone does not indicate the actual dietary contribution of *A. ursinum*, which also depends on the amount consumed and mineral bioavailability.

To provide a standardized nutritional interpretation, the mineral concentrations reported on a fresh-weight basis by Jędrszczyk et al. were expressed per 100 g of fresh leaves and compared with the EU Nutrient Reference Values (NRVs) for adults established by Regulation (EU) No 1169/2011 [63,72]. Accordingly, 100 g FW of wild garlic leaves would correspond to approximately 53.8% of the NRV for potassium, 12.6% for magnesium, 6.5% for iron, and 4.2% for calcium. These estimates should be interpreted cautiously, as 100 g represents a standardized reference quantity rather than an established habitual serving size for wild garlic. Moreover, the calculations are based on total mineral concentrations and do not account for gastrointestinal bioaccessibility or absorption.

Information on mineral bioavailability and antinutritional factors in *A. ursinum* remains limited. Oxalic acid has been detected in aqueous leaf extracts [73], but the available data are not expressed as absolute tissue concentrations and therefore do not allow assessment of its potential effect on mineral bioavailability. Similarly, species-specific quantitative information on phytates and other mineral-chelating constituents is currently insufficient. Processing may further influence the mineral content of *A. ursinum*-containing foods. In wild-garlic-enriched pasta, Filipčev et al. reported substantially lower retention of potassium after cooking (24.5–48.7%) than of Ca, Mg, Fe, and Zn, for which retention generally exceeded 80% [27].

In addition to essential mineral elements, nitrate accumulation is relevant when assessing the compositional quality of edible leafy plants. Čepulienė et al. reported a nitrate concentration of 43.1 ± 0.12 mg kg^−1^ FW in fresh *A. ursinum* leaves, providing complementary information on the compositional and food-quality characteristics of the edible plant material [67].

Overall, cross-study differences primarily reflect environmental variation, analytical methodology, and reporting basis. Although the reported concentrations demonstrate the presence of appreciable amounts of several essential macro- and microelements, their nutritional significance cannot be inferred from total concentrations alone. Actual dietary contribution depends on the amount consumed, processing-related losses, and mineral bioavailability, for which species-specific information remains limited. Therefore, future studies should combine standardized analytical and reporting approaches with assessments of mineral bioaccessibility and antinutritional factors to provide a more comprehensive evaluation of the nutritional contribution of *A. ursinum*.

### 5.4. Other Nutritionally Relevant Constituents

Beyond proximate composition, vitamin C and minerals, the nutritional profile of *Allium ursinum* L. includes additional constituents of nutritional relevance, although the available evidence is less comprehensive for these components. Carotenoids and several organic acids have been characterized, whereas information on individual sugars, folates and tocopherols remains comparatively limited.

Carotenoids represent an additional nutritionally relevant component of *A. ursinum*, particularly because its carotenoid fraction includes β-carotene, a precursor of vitamin A, together with lutein and other xanthophylls [43]. However, carotenoids also constitute an important class of specialized bioactive phytochemicals. Therefore, their detailed quantitative profile, distribution among plant organs and developmental stages, antioxidant relevance, and response to processing are discussed separately in Section 6.3.

Information on individual sugars remains comparatively limited. Available studies have predominantly quantified total carbohydrates, total sugars, soluble sugars or reducing sugars rather than providing comprehensive profiles of individual mono- and disaccharides; these data are therefore summarized in Section 5.1 and Table 2.

Organic acids constitute a less extensively characterized fraction of *A. ursinum.* GC-MS profiling of an aqueous extract prepared from dried leaves revealed glycolic, oxalic, malonic, benzoic, succinic, glyceric, malic, threonic, 2-oxoglutaric, shikimic, citric and galacturonic acids [73]. As the study primarily reported chromatographic signal areas rather than absolute concentrations of these organic acids, the results indicate their presence and relative representation in the extract but do not allow estimation of their quantitative dietary contribution.

In contrast to vitamin C and carotenoids, reliable species-specific quantitative data on folates and tocopherols in *A. ursinum* remain scarce in the available literature. Consequently, values reported for cultivated garlic or other *Allium* species were not extrapolated to *A. ursinum*. The limited characterization of these micronutrients represents an important gap in the current nutritional knowledge of the species and warrants targeted analysis in future studies.

Overall, the available evidence expands the nutritional characterization of *A. ursinum* beyond its proximate composition, vitamin C and mineral profile by documenting the occurrence of additional nutritionally relevant constituents. Nevertheless, the available evidence remains uneven across nutrient classes, particularly for individual sugars, organic acids, folates and tocopherols. Nutritional occurrence should also be distinguished from demonstrated biological activity: the presence or concentration of a compound in edible plant material does not by itself demonstrate a specific physiological or functional effect. Accordingly, sulfur-containing compounds, polyphenols, carotenoids, pigments and other specialized metabolites are examined in greater detail in Section 6 from the perspective of their phytochemical characteristics and potential functional food relevance.

## 6. Bioactive Compounds of *Allium ursinum* L. and Their Role in Functional Foods

The phytochemical profile of *Allium ursinum* L. comprises several major classes of specialized constituents, including sulfur-containing compounds, phenolic acids, flavonoids, carotenoids, chlorophylls, and other less extensively characterized compounds. The present section focuses on their chemical composition, distribution among plant organs, natural variability, processing stability, and relevance to the use of *A. ursinum* as a food ingredient. Evidence concerning their antioxidant, antimicrobial, cardiovascular, cytoprotective, and other biological activities is evaluated separately in Section 7.

Reported phytochemical profiles vary because of both biological factors and methodological differences in sample preparation, extraction, analysis, and data expression. [21,22,23].

**Figure 5:**
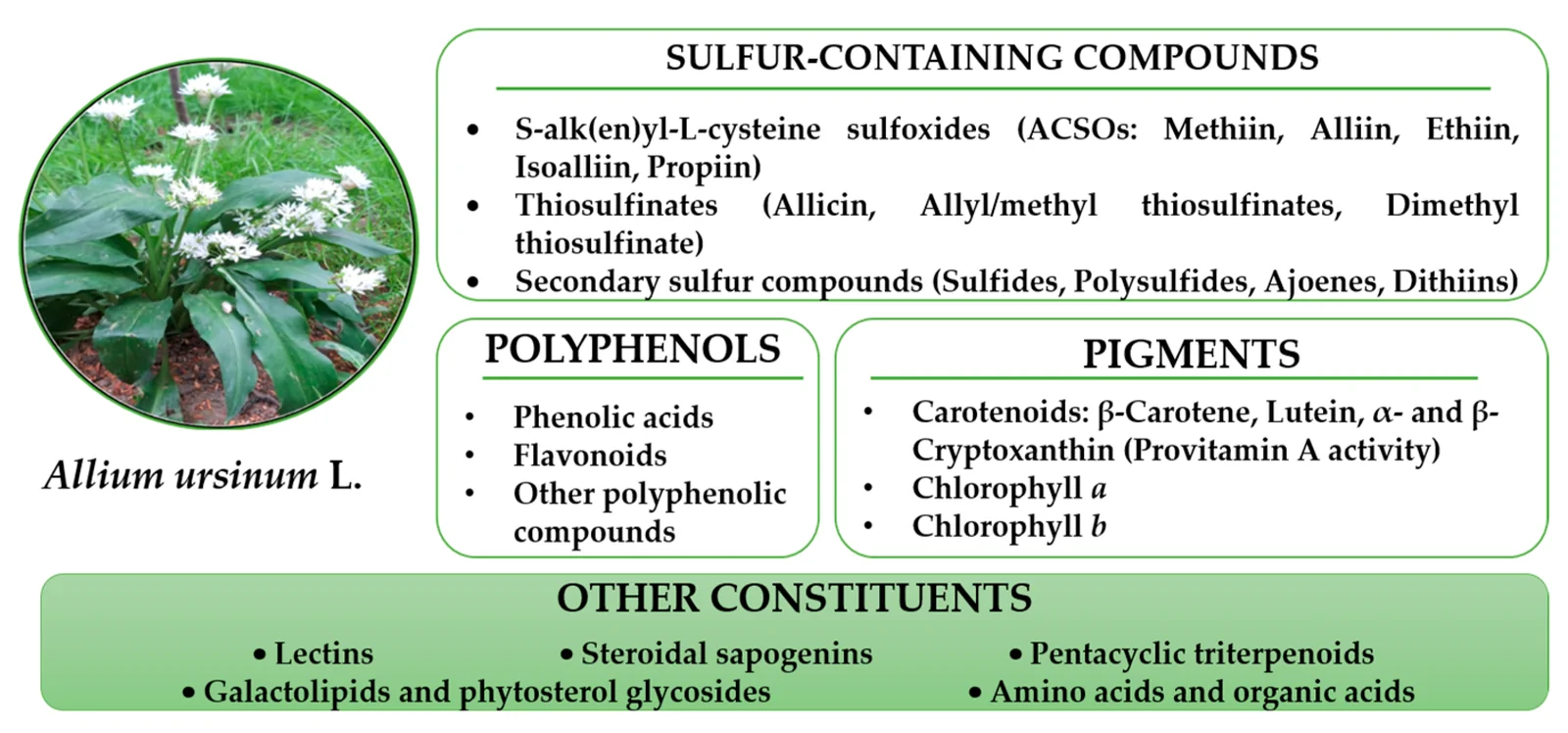
Major classes of bioactive compounds identified in *Allium ursinum* L. relevant to functional food applications.

Figure 5 provides a conceptual overview of the principal phytochemical classes reported in *A. ursinum* and their relevance to its compositional and food-related characteristics.

Among the various phytochemical groups, sulfur-containing compounds constitute the most characteristic and biologically distinctive constituents of *A. ursinum* and are particularly important for its sensory properties and processing behavior. Polyphenols and photosynthetic pigments constitute additional major components, whereas lectins, steroidal sapogenins, pentacyclic triterpenoids, galactolipids, phytosterol glycosides, amino acids, and organic acids remain less comprehensively characterized. Their composition, organ distribution, variability, and analytical determination are discussed in the following subsections.

To complement the classification-based overview presented in Figure 5, the chemical structures of representative compounds belonging to the two most extensively characterized phytochemical groups in *A. ursinum*-sulfur-containing compounds and flavonoids-are presented in Table 5. The selected structures illustrate the chemical diversity of these constituents and include the characteristic cysteine sulfoxide precursors alliin and methiin, the thiosulfinate allicin, the derived sulfur compounds diallyl disulfide, diallyl trisulfide and ajoene, as well as two representative kaempferol glycosides. Their inclusion is intended to facilitate the interpretation of the phytochemical profile rather than to indicate their universal quantitative predominance, as their abundance varies according to plant organ, developmental stage, origin and processing conditions.

### 6.1. Sulfur Compounds

Sulfur-containing compounds represent the most characteristic phytochemical class of *Allium ursinum* L. and are major determinants of its garlic-like aroma, sensory properties, and chemical behavior during food processing. In intact tissues, the sulfur profile is centered on non-volatile S-alk(en)yl-L-cysteine sulfoxides (ACSOs), which are spatially separated from alliinase. Methiin and alliin generally predominate, whereas ethiin, propiin, and isoalliin occur at lower or variable levels depending on plant organ, developmental stage, and geographical origin [7,15]. Representative chemical structures of the principal ACSO precursors and derived sulfur compounds are presented in Table 5.

Cutting, crushing, or other forms of tissue disruption bring ACSOs into contact with alliinase, which generates pyruvate, ammonia, and short-lived alk(en)yl sulfenic acids. These intermediates spontaneously condense into thiosulfinates, including allicin, allyl-methyl and methyl-allyl thiosulfinates, and dimethyl thiosulfinate. Because thiosulfinates are chemically unstable, they subsequently undergo non-enzymatic conversion into sulfides, polysulfides, ajoenes, and vinyl dithiins. Heating, drying, extraction, oxygen exposure, and storage therefore alter the relative abundance of these products and, consequently, the aroma and chemical stability of *A. ursinum* ingredients [5,15,82]. The principal sulfur-containing compounds reported in different organs, preparations, and extracts are summarized in Table 6.

Quantitative studies confirm that the sulfur profile is both species- and organ-dependent. Kubec et al. quantified methiin, alliin, ethiin, and propiin in fresh leaves, with methiin and alliin representing the principal ACSOs. Sendl and Wagner likewise found methiin and alliin in leaves and bulbs but reported differences in their amounts, expressed on a dry-weight basis, together with quantitative data for the corresponding thiosulfinates. These results distinguish the methyl- and allyl-rich precursor profile of *A. ursinum* from that of cultivated garlic, in which allyl-derived compounds generally predominate. However, direct comparison among studies remains limited by differences in plant material, preparation, analytical method, and reporting basis.

Following tissue disruption, ACSOs are rapidly hydrolyzed by alliinase, generating a complex mixture of thiosulfinates. Block et al. reported that allicin accounted for approximately 28 mol% of the thiosulfinate fraction, while the two allyl/methyl thiosulfinate isomers, AllS(O)SMe and AllSS(O)Me, accounted for 16 and 34 mol%, respectively. The substantial proportion of methyl-containing products is consistent with the abundance of methiin in *A. ursinum* tissues. These molar percentages describe the composition of the thiosulfinate profile obtained from the freeze-dried wild garlic powder extract and should not be interpreted as absolute concentrations in intact plant material.

Secondary sulfur products identified in *A. ursinum* include DADS, DATS, allyl methyl disulfide, allyl methyl trisulfide, dimethyl disulfide, dimethyl trisulfide, ajoenes, and vinyl dithiins. Gođevac et al., Ivanova et al., and Radulović et al. demonstrated considerable diversity in the volatile sulfur fraction, including methyl-, allyl-, and propyl-derived di-, tri-, and tetrasulfides. Kovačević et al. additionally found marked differences between the volatile profiles of bulbs and leaves collected from eight geographical locations. Because these studies used different sampling, extraction, and analytical procedures and generally expressed results as relative chromatographic abundances, their values represent matrix- and method-dependent profiles rather than directly comparable tissue concentrations.

Because thiosulfinates are highly reactive and chemically unstable, they rapidly decompose into a wide range of secondary sulfur metabolites, including sulfides, polysulfides, ajoenes and dithiin derivatives. As shown in Table 5, diallyl disulfide (DADS) and diallyl trisulfide (DATS) are among the major volatile sulfur compounds identified in essential oils and volatile extracts of *A. ursinum*. Studies based on GC-MS analysis further revealed the presence of allyl methyl disulfide, allyl methyl trisulfide, dimethyl disulfide and dimethyl trisulfide, demonstrating the considerable diversity of sulfur-containing volatiles detected in processed or extracted plant material.

The composition of volatile sulfur fractions is strongly influenced by sample preparation and postharvest treatment. Radulović et al. observed pronounced differences among fresh aerial parts, air-dried material, oven-dried material, and inflorescences, showing that sample preparation and drying conditions substantially affect the resulting volatile-oil composition. Consequently, the sulfur profile of a hydrodistilled essential oil cannot be considered equivalent to that of intact tissue. Instead, it reflects the combined effects of the native ACSO composition, enzymatic conversion following tissue disruption, and subsequent transformations during processing and analysis.

These transformations are directly relevant to food use because they influence garlic-like aroma, flavor intensity, sensory persistence, and the retention of chemically unstable thiosulfinates. Freshly disrupted material is characterized by rapidly generated thiosulfinates, whereas heating, drying, extraction, and storage shift the profile towards secondary sulfides, polysulfides, ajoenes, and vinyl dithiins. Thus, the technological treatment determines not only the sulfur profile but also the sensory characteristics and chemical stability of the resulting ingredient. These sulfur metabolites are also associated with the antimicrobial and antioxidant properties of *A. ursinum*, as discussed in Section 7.

### 6.2. Polyphenolic Compounds

Polyphenolic compounds represent one of the most important classes of non-sulfur bioactive constituents in *Allium ursinum* L. and contribute substantially to the phytochemical complexity of the species. Available chromatographic evidence indicates a flavonol profile dominated by glycosylated and acylated kaempferol derivatives, accompanied by phenolic acids occurring in free, bound, and conjugated forms. The principal phenolic acids reported include gallic, ferulic, vanillic, *p*-coumaric, and p-hydroxybenzoic acids. Total phenolic determinations and the major individual compounds identified in different plant organs are summarized in Table 7A,B, respectively [13,41,84,85,86].

From a quantitative perspective, leaves generally represent the richest source of total phenolic compounds, although considerable differences among plant organs have been reported. However, the available studies do not establish a uniform organ ranking under all experimental conditions. Kovačević et al., for example, reported relatively similar dry-weight total phenolic values for bulbs and leaves, whereas other studies found higher values in leaves than in flowers or bulbs. Differences have also been observed among populations and developmental stages within the same plant organ. Such differences reflect variation in plant population, developmental stage, sample preparation, extraction conditions, analytical procedure, and the basis used to express the results.

Cross-study comparison is limited by differences in plant material, extraction and hydrolysis procedures, analytical methodology, and reporting basis. Moreover, total phenolic content determined by the Folin-Ciocalteu assay provides an aggregate measure of reducing capacity rather than information on the identity or abundance of individual phenolic compounds. Chromatographic characterization is required to establish the identity and distribution of individual phenolic constituents. The major quantitative and qualitative profiles reported for *A. ursinum* are summarized in Table 7B.

Chromatographic analyses have shown that the phenolic composition of *A. ursinum* is dominated by flavonols and phenolic acids, with considerable variation among plant organs and extraction procedures.

Among the quantified individual compounds, gallic acid represents the most abundant phenolic acid reported in ramson leaves, reaching 75.8–322.5 mg/100 g in six Latvian accessions analyzed by Cinkmanis et al. In the same study, ferulic acid ranged from 8.3 to 47.8 mg/100 g, vanillic acid from 0.9 to 13.5 mg/100 g, and *p*-coumaric acid from 0.4 to 2.1 mg/100 g. These results indicate marked quantitative differences among the phenolic acids detected in the analyzed methanolic extracts. The same authors also quantified several flavonoids, including rutin (2.3–29.8 mg/100 g), catechin hydrate (0.1–23.6 mg/100 g) and epicatechin (2.4–12.3 mg/100 g), whereas quercetin, luteolin and kaempferol were detected chromatographically but could not be quantified reliably, illustrating the analytical complexity of the plant matrix [13].

Earlier studies demonstrated that quantification of extractable phenolics alone does not fully reflect the chemical complexity of the species. Djurdevic et al. demonstrated that phenolic acids occur in both free and bound fractions in leaves and bulbs. Ferulic and vanillic acids were detected in both fractions, whereas p-coumaric acid occurred at trace levels in the free fraction and was quantified in the bound fraction, while p-hydroxybenzoic acid was identified in the free bulb fraction. Vlase et al. further showed that the measured concentrations of p-coumaric and ferulic acids were higher after acid hydrolysis and that kaempferol was detected in *A. ursinum* leaves and flowers only after hydrolysis. Together, these studies demonstrate that the phenolic profile obtained analytically depends strongly on the extraction and hydrolysis procedures applied.

The structural diversity of flavonols represents one of the most remarkable phytochemical characteristics of *A. ursinum.* Carotenuto et al. and Wu et al. identified several flavonol glycosides, including previously unreported acetylated derivatives [90,91]. Oszmiański et al. subsequently demonstrated that kaempferol derivatives dominate the flavonoid fraction, reaching total concentrations of 1856.31 mg/100 g DW in green leaves and 2362.96 mg/100 g DW in yellow leaves. Leaves contained substantially higher concentrations than stalks or seeds, while differences between green and yellow leaves indicated that developmental and senescence-related changes affect the abundance of individual acylated derivatives.

Organ-dependent distribution was also demonstrated by Lachowicz et al., who identified 32 kaempferol derivatives and four caffeic acid derivatives in different anatomical parts. Leaves contained the greatest overall abundance of identified phenolics, followed by flowers and stems, whereas roots contained the lowest total amount but displayed a distinct profile in which caffeic acid derivatives were detected. This distribution indicates that the phenolic profile varies not only quantitatively but also qualitatively among plant organs.

Overall, *A*. *ursinum* shows a kaempferol-dominated flavonol profile accompanied by phenolic acids occurring in different extractable and bound forms, depending on the compound and analytical procedure. Their contribution to the antioxidant properties of *A*. *ursinum* is discussed in Section 7.1.

### 6.3. Pigments and Other Antioxidant Phytochemicals

Besides sulfur-containing compounds and polyphenols, *Allium ursinum* L. contains photosynthetic pigments, particularly chlorophylls and carotenoids, that contribute to its broader phytochemical profile. In food systems, their relevance depends not only on their initial abundance but also on their chemical stability, release from plant tissues, interactions with the food matrix, and accessibility during digestion.

The principal carotenoids and chlorophylls reported for the species are summarized in Table 8 [22,41,43,66,70,71].

The available evidence demonstrates marked organ-dependent differences in the pigment composition of *A*. *ursinum*. Lachowicz et al. reported markedly higher total carotenoid concentrations in leaves than in stems, flowers, or bulbs, confirming that photosynthetically active tissues are the main sites of pigment accumulation. The leaf carotenoid profile included all-trans-β-carotene, all-trans-lutein, α-cryptoxanthin, β-cryptoxanthin, and 9-cis-β-carotene. Radušienė et al. likewise detected lutein in leaves and flowers, although its abundance varied substantially among populations and was consistently higher in leaves [41].

Pigment abundance varies across organs, developmental stages, and environmental conditions. Lachowicz et al. observed differences between March and June harvests, while Voća et al. found differences in total chlorophyll and carotenoid contents between leaves collected before flowering and during the flowering stage. Gordanić et al. reported substantial variation among plants growing under different soil conditions, and Radušienė et al. documented population-dependent differences in lutein content. These studies collectively indicate that both biological origin and environmental context influence the pigment profile of *A*. *ursinum* [41,70,71].

Additional evidence for the importance of pigments in *A*. *ursinum* leaves was provided by Luță et al., who reported 174.49 ± 17.09 mg 100 g^−1^ FW total chlorophylls and 5.29 ± 0.41 mg 100 g^−1^ FW total carotenoids in fresh Romanian wild garlic leaves. Although these values were obtained using spectrophotometric methods and are therefore not directly comparable with chromatographic determinations of individual pigments, they further confirm the substantial accumulation of photosynthetic pigments in the edible aerial parts of the species [66].

Postharvest processing further modifies pigment composition. Roșan et al. observed lower measured chlorophyll concentrations in frozen extracts than in extracts obtained from fresh leaves, whereas drying produced higher concentrations when results were expressed per milliliter of extract. Similarly, pigment values reported for frozen, air-dried, and freeze-dried material varied according to both treatment and the basis used for expression. Such apparent increases after drying should not be interpreted as postharvest pigment synthesis; they may reflect water removal, concentration of dry matter, altered extraction efficiency, or greater release of pigments from disrupted tissues. At the same time, processing may promote pigment degradation, isomerization, oxidation, or conversion of chlorophylls into derivatives, meaning that measured concentration alone does not fully describe pigment stability [22].

Chromatographic and spectrophotometric methods provide different types of information, and results expressed on fresh-weight, dry-weight, or extract-volume bases are therefore not directly comparable. Chromatographic approaches enable identification and quantification of individual carotenoids, whereas spectrophotometric methods generally estimate total chlorophyll or carotenoid fractions.

Although chlorophylls and carotenoids may contribute to the overall antioxidant profile of *A*. *ursinum*, the available evidence does not establish that their contribution to its biological effects is equivalent to that of sulfur compounds or polyphenols. Processing may influence not only pigment stability but also their release from the plant matrix and potential bioaccessibility. However, no species-specific digestion, absorption, or bioavailability studies were identified for carotenoids or chlorophylls derived from *A*. *ursinum*. This represents an important research gap, particularly for the development of processed foods containing wild-garlic ingredients. Their contribution to the antioxidant properties of *A*. *ursinum* is discussed in Section 7.1.

### 6.4. Other Bioactive Compounds

In addition to sulfur compounds, polyphenols and photosynthetic pigments, *Allium ursinum* L. contains several other constituents that increase its phytochemical diversity. These include mannose-specific lectins, steroidal sapogenins and glycosides, pentacyclic triterpenoids, galactolipids, phytosterol glycosides, amino acids, and organic acids. For most of these constituents, the available evidence concerns chemical identification, structural characterization, isolation, or targeted quantification and should not, by itself, be interpreted as evidence of biological efficacy in foods or humans.

Table 9 summarizes these constituents, their organ distribution, sample conditions, analytical methods, and quantitative data when available. Because the reported values represent different analytical endpoints-including tissue concentrations, extract-related percentages, isolation yields, and relative chromatographic signals-they are not directly comparable.

The available evidence indicates marked organ-dependent variation. Sobolewska et al. found substantially higher diosgenin levels in bulbs than in leaves, with the highest values recorded before flowering. However, because diosgenin was measured after acid hydrolysis, the reported values represent released sapogenin rather than individual intact steroidal glycosides. Lachowicz et al. identified betulinic, oleanolic, and ursolic acids in all investigated organs, with total triterpenoid concentrations being highest in leaves and lowest in bulbs and varying between the March and June collections.

Sabha et al. isolated the galactolipid DLGG and β-sitosterol-3-O-β-D-glucopyranoside from ethanolic leaf extracts. The reported amounts-2.23 g of isolated DLGG, corresponding to approximately 1.26% of the dry ethanolic extract, and 17 mg of the phytosterol glucoside-represent isolation yields or extract-related abundance rather than concentrations in the original plant material. Accordingly, these values cannot be compared directly with the tissue-based concentrations reported for diosgenin or triterpenoids. Biological evidence associated with these lipophilic constituents is discussed in Section 7.

Kovačević et al. quantified 14 amino acids in bulbs and leaves collected from eight Croatian populations. Arginine predominated in bulbs, whereas glutamic acid predominated in leaves, and the profiles varied considerably among collection locations. Hangweirer et al. tentatively identified 12 organic acids in a hot-water extract prepared from dried leaves. Because these identifications were based on mass-spectral and retention-index matching and were expressed as relative chromatographic peak areas, they demonstrate the qualitative diversity of the organic-acid fraction but do not provide absolute tissue concentrations.

Mannose-specific lectins constitute the most extensively characterized group at the molecular level. The bulb lectins AUAI and AUAII have been investigated with respect to their structure, biosynthesis, genetic organization, and post-translational processing, whereas the structurally distinct lectin AUAL has been isolated and characterized from leaves [92,93,94,95]. Despite this detailed molecular characterization, quantitative concentrations expressed on a fresh- or dry-weight basis remain unavailable. Moreover, AUAI-alliinase complexes isolated from bulb extracts may form immediately after tissue homogenization and therefore should not be assumed to occur as pre-existing complexes in intact tissues [93].

Overall, the phytochemical profile of *A*. *ursinum* reflects the combined influence of plant organ, developmental stage, geographical and environmental conditions, processing, extraction procedure, analytical methodology, and reporting basis. These factors contribute substantially to the variability observed across studies and should be considered when comparing compositional and biological data. At the same time, the combined presence of organosulfur compounds, polyphenols, pigments, and other specialized constituents provides the biochemical basis for the biological activities discussed in Section 7.

## 7. Biological Activities Supporting Functional Food Potential

Experimental studies indicate that these constituents may act synergistically and contribute to the biological potential of *A*. *ursinum* L. through antioxidant, antimicrobial, cardiovascular, and cytoprotective effects. However, most of the available evidence originates from chemical assays, in vitro systems, cellular models, and animal experiments, whereas controlled human intervention and human pharmacokinetic studies remain very limited. Accordingly, the evidence discussed in this section is interpreted according to the experimental level at which it was obtained, distinguishing chemical or cell-free assays, cellular and other in vitro models, animal studies, food-matrix investigations, and human evidence. These evidence categories should not be regarded as equivalent in terms of biological or clinical relevance. Accordingly, experimental findings should not be extrapolated directly to health effects in consumers or to validated efficacy in functional foods [96].

**Figure 6:**
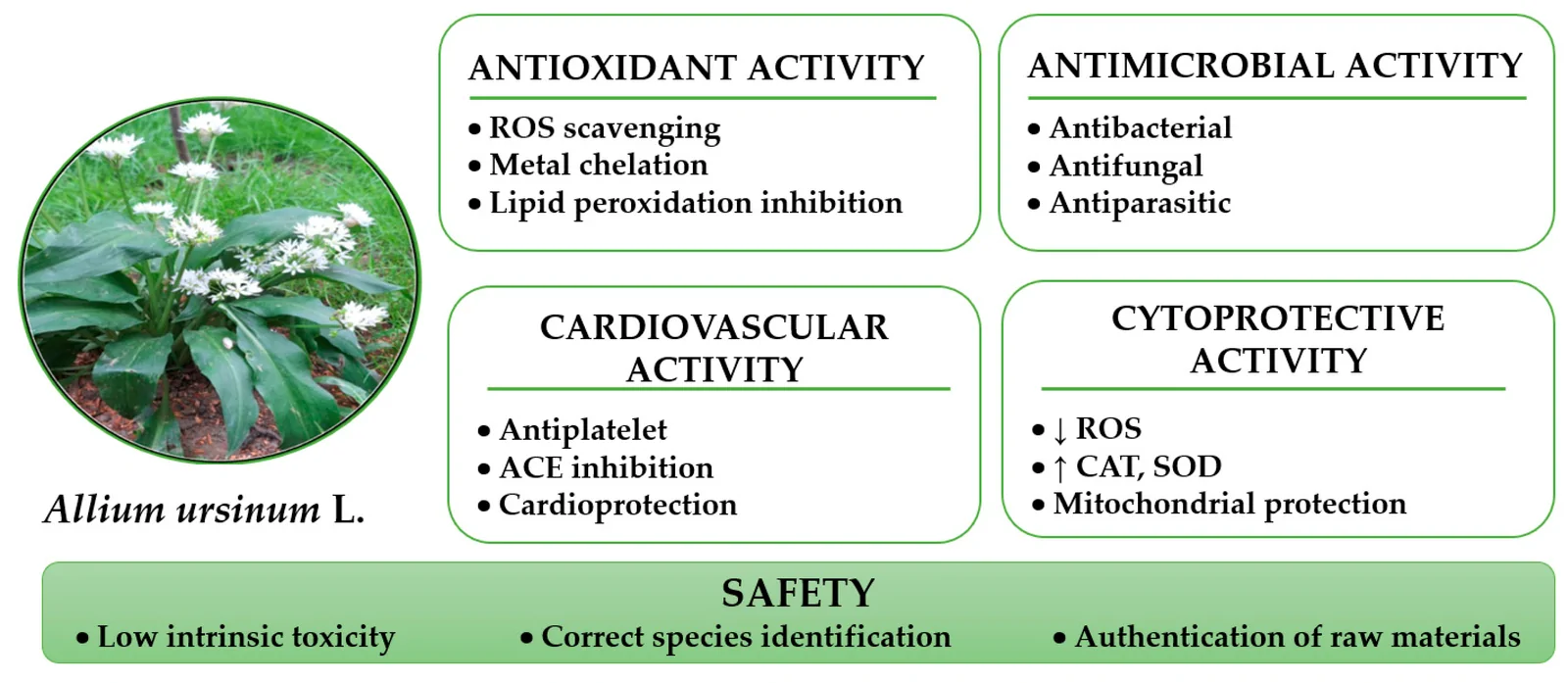
Overview of the major biological activities of *Allium ursinum* L. relevant to functional food applications. ↑ indicates an increase, whereas ↓ indicates a decrease.

As illustrated in Figure 6, the biological activities reported for *A*. *ursinum* reflect its complex phytochemical composition but vary with plant material, extract composition, dose, experimental model, food matrix, and investigated endpoint. Their relevance to functional food applications should therefore be interpreted in relation to the limitations of the available evidence and the safety requirements associated with correct identification, authentication, and intended use.

Among these constituents, organosulfur compounds such as alliin, allicin and their transformation products are considered major contributors to the antimicrobial, antifungal and antiparasitic properties of *A*. *ursinum* [5,15,82]. In parallel, polyphenolic compounds, particularly flavonoid glycosides and kaempferol derivatives, contribute substantially to antioxidant activity and have been associated with additional biological effects, including inhibition of platelet aggregation and potential cardiovascular benefits [25,86,91,97].

The growing interest in plant-derived bioactive compounds is driven by their relevance in functional food development, where antioxidant and antimicrobial properties contribute to both product stability and potential health benefits [70,98,99]. In this context, *A*. *ursinum* represents a promising natural source of multifunctional ingredients with applications in food, nutraceutical and pharmaceutical systems.

Consequently, the biological activities reported in the literature should be interpreted within the context of the experimental conditions employed rather than as fixed properties of the species.

This section provides a comprehensive overview of the major biological activities of *A*. *ursinum* relevant to functional food applications, including antioxidant, antimicrobial, cardiovascular, and cytoprotective effects, together with key safety considerations associated with its use.

### 7.1. Antioxidant Capacity

The antioxidant capacity of *Allium ursinum* L. has been extensively investigated using various in vitro assays, including DPPH, FRAP, ABTS, ORAC, and antioxidant enzyme-based evaluations, as summarized in Table 10. Early studies demonstrated measurable antioxidant capacity in extracts from different plant organs, with substantial organ- and assay-dependent variation [100]. Most of the evidence summarized in Table 10 represents chemical antioxidant capacity measured in cell-free assay systems. These assays evaluate distinct chemical mechanisms and should not be considered interchangeable: DPPH and ABTS/TEAC assess radical-quenching capacity through reaction mechanisms involving electron and/or hydrogen-atom transfer, FRAP measures reducing capacity, whereas ORAC primarily estimates peroxyl-radical scavenging through hydrogen-atom transfer. H_2_O_2_-scavenging and related assays likewise represent specific chemical reactivity and should not be interpreted as direct evidence of antioxidant efficacy in biological systems [101,102]. These results should be distinguished from antioxidant effects demonstrated in cellular or animal models, which are discussed separately where available. Because DPPH, FRAP, ABTS, and ORAC rely on different reaction mechanisms and are often expressed in different units, their results should be interpreted comparatively rather than directly compared across studies.

The considerable variability in antioxidant capacity reported for *A*. *ursinum* (Table 10) is attributable to both biological and methodological factors. From a biological perspective, antioxidant compounds are unevenly distributed among plant organs. Leaves have shown high antioxidant capacity in several comparative studies of *A*. *ursinum*, although the organ exhibiting the highest activity depends on the assay and phytochemical profile. The high antioxidant activity reported for leaves has been associated with antioxidant enzyme activity and with the presence of antioxidant phytochemicals, including carotenoids and chlorophylls [43,100].

However, organ-specific differences may vary depending on the antioxidant assay employed. Lupoae et al., who evaluated leaves, flowers, roots and bulbs using DPPH, TEAC, H_2_O_2_ scavenging, NO scavenging and electrochemical methods, reported the highest DPPH radical scavenging activity in flower extracts (65.69%), whereas fresh roots exhibited the strongest H_2_O_2_ scavenging activity [106]. These endpoints represent different chemical reactivities and should therefore be interpreted separately rather than combined into a single measure of antioxidant efficacy. These findings indicate that different classes of antioxidants may predominate in different plant organs and respond differently depending on the analytical method used to assess antioxidant capacity. Nevertheless, leaves consistently remain among the most important reservoirs of antioxidant phytochemicals in *A*. *ursinum*.

Phenological development also contributes significantly to antioxidant variability. Tóth et al. demonstrated increasing DPPH scavenging activity during plant maturation, while Voća et al. observed substantial differences between vegetative and flowering stages. Such changes are likely associated with seasonal shifts in secondary metabolism, as plants synthesize protective metabolites in response to increasing solar radiation, reproductive development, and environmental stress. Similar conclusions were reached by Radušienė et al., who showed that climatic factors, particularly precipitation and spring temperature, significantly influence phytochemical accumulation and antioxidant performance in natural populations.

The extraction procedure represents another major source of variation. Antioxidant compounds in *A*. *ursinum* differ considerably in polarity, chemical stability, and solubility. Consequently, aqueous, alcoholic, pressurized-liquid, and chloroform extracts recover different groups of bioactive molecules, resulting in substantially different antioxidant responses [103,105]. Tomovic et al. [105] reported IC_50_ values of 111.04, 154.25, and 391.79 µg mL^−1^ for methanolic, aqueous, and chloroform leaf extracts, respectively. Because lower IC_50_ values indicate stronger DPPH radical-scavenging activity, the methanolic extract showed the highest activity, whereas the chloroform extract was the least active. These differences may partly reflect the selective extraction of compounds with different polarity and radical-scavenging properties; however, the contribution of individual phytochemical classes cannot be established from these data alone.

Additional evidence for the multifactorial nature of antioxidant activity was provided by Luță et al., who reported a DPPH EC_50_ value of 17.39 ± 1.46 mg mL^−1^ for fresh *A*. *ursinum* leaf extracts. More importantly, the authors demonstrated strong correlations between antioxidant activity and the concentrations of phenolic compounds, flavonoids, chlorophylls, and carotenoids. Among the investigated phytochemicals, flavonoids exhibited the strongest association with radical scavenging capacity, whereas vitamin C showed only a moderate correlation. These findings indicate that the antioxidant activity of *A*. *ursinum* results from the combined contribution of multiple phytochemical groups rather than from a single dominant antioxidant constituent [66].

Processing treatments further influence antioxidant measurements. Roșan et al. attributed the higher DPPH and FRAP values of dehydrated leaves mainly to water removal, whereas Bernaś et al., reporting results on a dry-matter basis, also found significant differences among preservation methods. These findings indicate that processing effects cannot be explained by water loss alone and may also involve treatment-specific changes in antioxidant constituents, including substantial vitamin C degradation during air-drying.

Methodological differences among antioxidant assays constitute an additional source of variability. Because different phytochemicals react preferentially in different assay systems, extracts showing high activity in one method do not necessarily exhibit proportional activity in another. This phenomenon explains why antioxidant values reported across studies often differ substantially even when similar plant materials are analyzed.

Therefore, the variability observed among studies should not be interpreted as inconsistency but rather as evidence of the complex and multifactorial nature of antioxidant activity in *A*. *ursinum*, which depends on phytochemical composition, plant physiology, environmental conditions, extraction strategy, processing treatment, and analytical methodology.

Overall, the available evidence should be interpreted according to the endpoint assessed. DPPH, ABTS/TEAC, FRAP, ORAC, H_2_O_2_-scavenging, and related tests characterize distinct aspects of chemical antioxidant capacity, whereas inhibition of lipid oxidation, modulation of endogenous antioxidant enzymes, cellular ROS responses, and in vivo oxidative-stress biomarkers provide progressively more biologically relevant evidence. These categories are complementary but not interchangeable.

### 7.2. Antimicrobial and Antifungal Activities

The antimicrobial activity of *Allium ursinum* L. has attracted considerable attention due to its potential applications in food preservation, functional foods, and complementary antimicrobial therapies. The biological activity of wild garlic is mainly attributed to sulfur-containing compounds, particularly allicin and related thiosulfinates, which can react with thiol-containing enzymes and proteins, thereby disrupting essential microbial metabolic pathways [107]. In addition to organosulfur compounds, polyphenols and flavonoids may contribute to the overall antimicrobial effect through membrane destabilization and oxidative stress modulation.

The available studies demonstrate that *A*. *ursinum* exhibits activity against a broad spectrum of microorganisms, including Gram-positive and Gram-negative bacteria, yeasts, and filamentous fungi. However, antimicrobial efficacy varies considerably depending on plant organ, extraction procedure, solvent polarity, target microorganism, and analytical method. Quantitative data reported in the literature are summarized in Table 11.

The quantitative data summarized in Table 11 demonstrate substantial variability in antimicrobial activity depending on extraction procedure, microbial strain, plant organ, and assay employed. Overall, yeasts and filamentous fungi generally appeared more susceptible than bacteria. For example, *Candida* spp. were inhibited at concentrations of 0.5–4.0 mg/mL, whereas substantially higher concentrations were required to inhibit bacterial growth [108]. Similarly, Stupar et al. reported MIC and MBC values of 28–30 mg/mL for the investigated foodborne bacteria and further showed that antibacterial efficacy decreased as the initial microbial load increased.

One of the main sources of variability among studies is the extraction procedure. Organosulfur compounds, phenolics and flavonoids differ in polarity and stability; therefore, aqueous, hydroalcoholic, ethanolic and organic solvent extracts recover different bioactive constituents. Accordingly, Ivanova et al., Lupoae et al. and Pavlović et al. reported marked differences in antimicrobial activity among extracts obtained with different solvents. Biological variability also contributes to these differences. Flower extracts generally exhibited stronger antifungal activity than leaf extracts because of their higher allicin content [112], whereas Burton et al. demonstrated that allicin was the only metabolite significantly associated with antibacterial activity, supporting the central role of sulfur compounds in the antimicrobial properties of *A*. *ursinum*.

Direct comparison among studies should nevertheless be made with caution. Antimicrobial activity was assessed using different methodologies, including broth microdilution, agar diffusion and food-model experiments, which are not directly comparable. Moreover, allicin is highly unstable and rapidly degrades after tissue disruption, making antimicrobial activity strongly dependent on extraction conditions and sample handling. Finally, food-matrix studies demonstrated considerably lower antimicrobial efficacy than in vitro assays [114,115], indicating that interactions with proteins, lipids and other food components may reduce the accessibility and effective antimicrobial concentration of active compounds within the food matrix.

Overall, antimicrobial efficacy is highly context-dependent and remains more consistently demonstrated in vitro than in food matrices. No clinical studies demonstrating antimicrobial efficacy of *A*. *ursinum* were identified; therefore, in vitro susceptibility data and food-preservation effects should not be extrapolated to therapeutic antimicrobial activity in humans.

### 7.3. Cardiovascular Activity

Cardiovascular diseases remain a leading cause of mortality worldwide, increasing interest in natural compounds capable of modulating multiple cardiovascular risk factors. Experimental studies indicate that *A*. *ursinum* L. exerts antihypertensive, antiatherogenic, antiaggregatory, antioxidant, antiarrhythmic, and cardioprotective effects through the combined action of sulfur-containing compounds, phenolics, sterols, and other bioactive metabolites [20,24,31,116,117,118,119]. The cardiovascular effects reported for *A*. *ursinum* are summarized in Table 12.

The cardiovascular evidence spans several experimental levels, including in vitro assays using human platelets, cellular models, and predominantly animal studies. No controlled human cardiovascular intervention studies were identified; therefore, the findings summarized below should be interpreted as preclinical or mechanistic evidence rather than as demonstration of clinical cardiovascular efficacy.

As shown in Table 12, the cardiovascular activity of *A*. *ursinum* involves several complementary mechanisms. Early experimental studies demonstrated that dietary wild garlic markedly reduced susceptibility to ischemia- and reperfusion-induced ventricular arrhythmias. Rietz et al. reported a reduction in ventricular fibrillation incidence from 88% to 20% during ischemia and from 90% to 50% during reperfusion, accompanied by a significant decrease in the size of the ischemic zone. The authors also identified a moderate ACE-inhibitory activity that may contribute to the observed cardioprotective effects.

Antihypertensive and lipid-lowering effects were subsequently demonstrated by Preuss et al., who observed reductions in systolic blood pressure and total cholesterol, accompanied by increased HDL concentrations in spontaneously hypertensive rats. These findings support the traditional use of *A*. *ursinum* in cardiovascular disorders.

Another important mechanism is the inhibition of platelet aggregation. In vitro assays using human platelets, alcoholic leaf extracts significantly reduced ADP-induced and, to a lesser extent, epinephrine-induced platelet aggregation [24]. Further fractionation studies identified a galactolipid and a phytosterol as active antiaggregatory constituents, indicating that the cardiovascular effects of *A*. *ursinum* cannot be attributed solely to sulfur compounds [25].

Strong antiatherogenic effects were reported in hypercholesterolemic rabbits. Administration of leaf lyophilizate reduced LDL and ApoB concentrations, decreased aortic plaque formation, improved cardiac performance, and increased HO-1 expression, suggesting activation of endogenous cytoprotective pathways. These findings were consistent with earlier preliminary observations reported by the same research group, which demonstrated beneficial effects of *A*. *ursinum* herbal extract in hypercholesterolemic hearts [118]. In an animal model of pulmonary hypertension, the same preparation improved right ventricular function and reduced pulmonary vascular remodeling, producing effects comparable to sildenafil [119].

The cardioprotective role of *A*. *ursinum* is further supported by studies addressing oxidative stress. In a cellular model of doxorubicin-induced cardiotoxicity, AU water extract reduced intracellular and mitochondrial ROS generation [31]. Furthermore, chronic administration of methanolic *A*. *ursinum* extract (125–500 mg kg^−1^ for 28 days) significantly improved recovery of myocardial function following ischemia-reperfusion injury, as reflected by enhanced cardiac contractility, systolic function, coronary flow, and attenuation of oxidative stress markers [20]. The highest dose produced the most pronounced cardioprotective and antioxidant effects.

Although sulfur-containing compounds are generally regarded as the principal active constituents, the available evidence indicates that the observed cardiovascular effects result from synergistic interactions among sulfur metabolites, phenolics, sterols, and lipid-derived compounds. Nevertheless, most data originate from animal or cellular models, and clinical studies in humans remain limited. Despite this limitation, the consistency of experimental findings supports the potential of *A*. *ursinum* as a functional food ingredient and complementary strategy for cardiovascular health promotion.

Overall, experimental studies indicate that *A*. *ursinum* may exert multifactorial cardioprotective effects, including antihypertensive, antiaggregatory, antioxidant, antiatherogenic, and antiarrhythmic activities. These findings support its further investigation as a potential ingredient for cardiovascular-oriented functional foods and nutraceuticals. However, because the available evidence originates predominantly from cellular and animal models, these effects should not be interpreted as demonstrating clinical efficacy or cardiovascular health benefits in humans.

### 7.4. Cytoprotective Effects and Potential Anti-Inflammatory Activity

Biological redox effects reported in these studies include modulation of endogenous antioxidant defenses (CAT, SOD, HO-1), reduction of lipid-oxidation markers (MDA, TBARS), and attenuation of intracellular or mitochondrial ROS.

Available experimental evidence indicates that *Allium ursinum* L. exerts cytoprotective effects primarily through attenuation of oxidative stress rather than through direct modulation of inflammatory signaling. The available evidence derives from ex vivo human-cell systems, cultured cardiomyoblasts, and animal models; no controlled human intervention studies directly evaluating cytoprotective or anti-inflammatory effects were identified. As summarized in Table 13, most studies evaluated lipid peroxidation, reactive oxygen species (ROS), antioxidant enzymes or stress-response proteins, whereas only limited information is available regarding canonical inflammatory mediators such as TNF-α, IL-1β, IL-6, NF-κB, COX-2 or iNOS. Consequently, the current evidence supports cytoprotective and redox-modulating activity with greater confidence than direct anti-inflammatory effects.

The studies summarized in Table 13 consistently demonstrate protection against oxidative injury, although the magnitude of the response depends on the experimental model and extract composition. Cellular oxidative-stress evidence is provided mainly by H9c2 cardiomyoblast model, in which aqueous leaf extract reduced intracellular and mitochondrial ROS generation but did not improve cell viability. Davidović-Plavšić et al. provided the most direct evidence of membrane protection by showing that a phenolic leaf extract significantly reduced malondialdehyde accumulation and hemoglobin oxidation while increasing catalase activity in human erythrocytes exposed to terbuthylazine. In contrast, Pop et al. demonstrated that cytoprotective activity was strongly extract-dependent. Only the aqueous leaf extract reduced intracellular and mitochondrial ROS, whereas neither extract protected H9c2 cardiomyoblast viability.

Comparable observations were obtained in vivo. Ranković et al. showed that methanolic extract of the aerial parts reduced TBARS and other pro-oxidative markers while enhancing CAT and SOD activities after myocardial ischemia/reperfusion injury, resulting in improved post-ischemic cardiac recovery. Likewise, Bombicz et al. reported increased HO-1 expression together with reduced SOD-1 protein levels, lower LDL and ApoB concentrations, and reduced atherosclerotic plaque formation in hypercholesterolemic rabbits. Rather than representing contradictory findings, the apparent differences among studies largely reflect the use of distinct biological models, oxidative stress markers and experimental endpoints. Whereas erythrocyte and cell culture models primarily evaluate direct protection against oxidative injury, animal studies assess tissue adaptation through activation of endogenous defense mechanisms.

At the in vivo level, animal studies reported changes in oxidative-stress biomarkers, including reduced TBARS and altered antioxidant-enzyme activity, which provide a different level of evidence from cell-free antioxidant assays.

Taken together, the available evidence supports cytoprotective and redox-modulating activity more strongly than direct anti-inflammatory activity. Reductions in reactive oxygen species, lipid peroxidation, or tissue injury may suggest an indirect anti-inflammatory potential associated with improved redox homeostasis, but they should not be interpreted as demonstrating direct modulation of inflammatory pathways. Confirmation of anti-inflammatory activity therefore requires studies that directly evaluate inflammatory cytokines and canonical signaling mediators, including TNF-α, IL-1β, IL-6, NF-κB, COX-2, and iNOS.

### 7.5. Proposed Mechanism of Action

Taken together, the available evidence suggests that the biological potential of *A*. *ursinum* L. reflects the integrated contribution of organosulfur compounds, polyphenols, flavonoids, and other bioactive constituents through complementary antimicrobial, redox-modulating, and cardiovascular pathways. Organosulfur metabolites are primarily associated with disruption of microbial enzymes and membranes, whereas sulfur-containing compounds and phenolics contribute to redox homeostasis through radical-scavenging mechanisms and modulation of endogenous antioxidant defenses [25,26,31,91]. Cardiovascular effects appear to involve antiaggregatory, ACE-related, and vascular mechanisms, with additional contributions from galactolipids and phytosterols [24,25,116,117,119]. Across different experimental models, these pathways converge on cytoprotective and cardioprotective outcomes, although direct interactions among phytochemical classes have not been experimentally established. The proposed model should therefore be regarded as an integrative mechanistic framework rather than as a validated unified mechanism.

**Figure 7:**
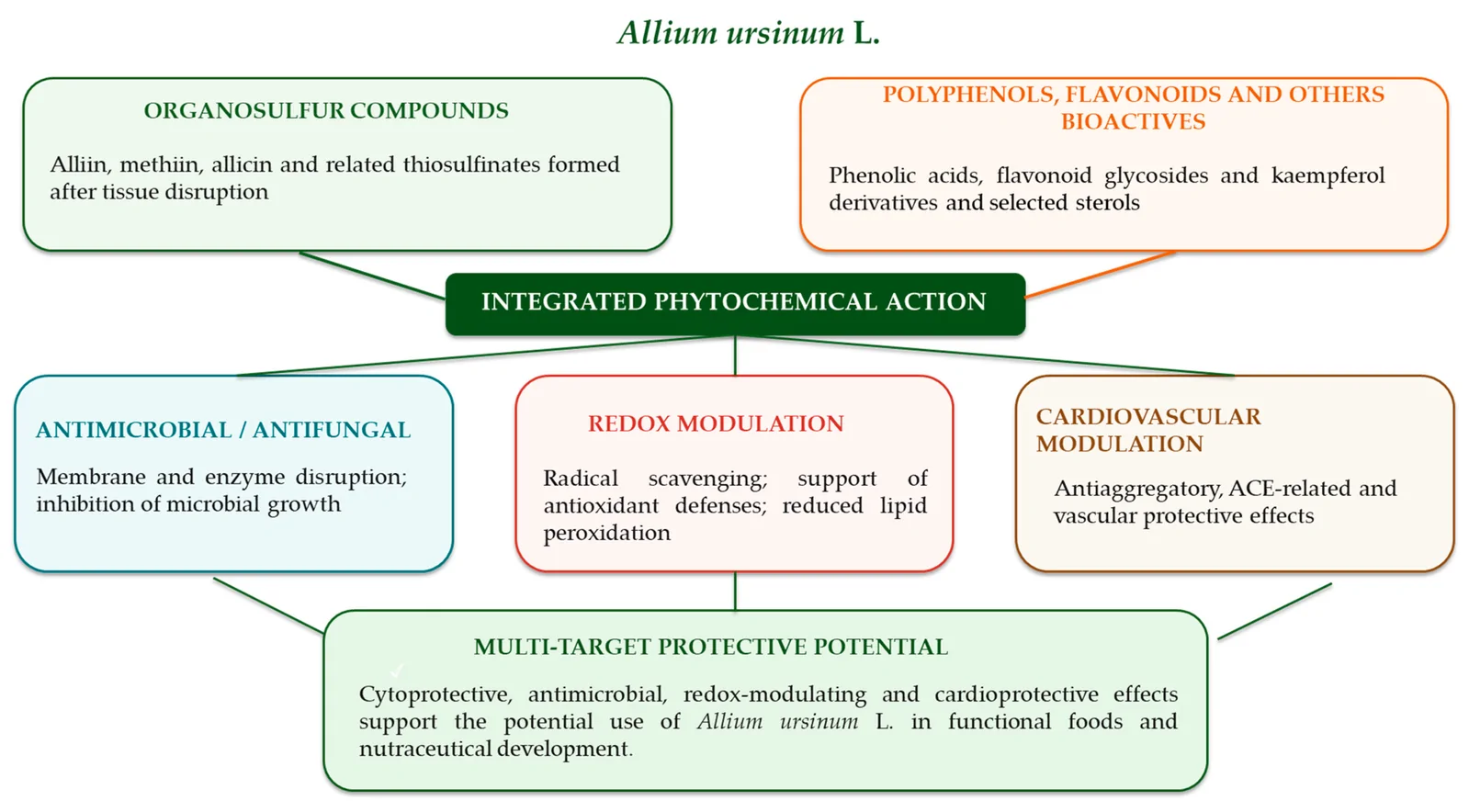
Proposed integrative mechanism of action of *Allium ursinum* L., linking phytochemical composition to antimicrobial, redox and cardiovascular pathways.

As illustrated in Figure 7, these complementary mechanisms provide a concise framework linking the phytochemical composition of *A*. *ursinum* with its antimicrobial, redox-modulating, and cardiovascular effects and its potential for functional food applications.

### 7.6. Toxicological Profile and Safety Considerations

Unlike many medicinal plants, the principal safety concern associated with *Allium ursinum* L. is not a demonstrated intrinsic toxicity of the correctly identified edible species, but the recurrent risk of accidental misidentification with poisonous plants that share similar morphology and ecological habitats. The greatest risk is associated with confusion with *Colchicum autumnale* and *Convallaria majalis*, particularly during early spring when leaves are harvested before flowering and diagnostic reproductive structures are absent [32,120].

The toxicological significance of these species differs considerably. *Colchicum autumnale* contains colchicine, a highly toxic alkaloid that may cause life-threatening intoxication, whereas *Convallaria majalis* contains cardiac glycosides, including convallatoxin, which induce digitalis-like toxicity [30,32]. Clinical manifestations associated with colchicine poisoning include gastrointestinal symptoms occurring within 4–12 h after ingestion, followed by potentially severe complications such as renal failure, cardiovascular collapse, respiratory insufficiency, multiorgan dysfunction, and death [30]. The principal toxicological risks associated with wild garlic harvesting are summarized in Table 14.

As shown in Table 14, the toxicological hazard associated with wild garlic is primarily indirect and results from accidental substitution. Rousseau et al. described a clinical case of colchicine poisoning after ingestion of leaves collected as wild garlic. The patient developed gastrointestinal symptoms within hours after consumption, followed by thrombocytopenia and hepatic cytolysis, confirming that confusion with *C*. *autumnale* remains a relevant public health concern. Similarly, Vollmer et al. emphasized that ingestion of colchicine doses exceeding 0.5 mg/kg may be associated with high fatality rates and highlighted the need for rapid analytical confirmation in suspected poisoning cases.

Recent investigations have significantly improved the ability to distinguish *A*. *ursinum* from toxic adulterants. M-Hamvas et al. demonstrated that *A*. *ursinum* leaves are hypostomatic, with predominantly abaxial stomata, whereas both *C*. *majalis* and *C*. *autumnale* are amphistomatic. Furthermore, only *A*. *ursinum* possesses characteristic wavy epidermal cell walls on the abaxial surface, while the toxic species exhibit straight cell walls. These features remain detectable even in dried plant material and therefore provide valuable tools for food authentication and forensic identification.

Because accidental substitution of *A*. *ursinum* with morphologically similar toxic species remains the principal safety concern, reliable authentication methods are essential for ensuring consumer safety. In addition to anatomical and microscopic approaches, metabolomic techniques have emerged as powerful tools for detecting adulteration and supporting species authentication. Ivanović et al. demonstrated that untargeted metabolomic fingerprinting combined with multivariate analysis can successfully differentiate *A*. *ursinum* from poisonous adulterants. Specific biomarkers such as isovitexin, vicenin II, azetidine-2-carboxylic acid, and trigonelline were identified as indicators of contamination with *Convallaria majalis*, whereas isovitexin also served as a marker of adulteration with *Arum maculatum*. The principal authentication approaches currently available are summarized in Table 15.

For food and nutraceutical applications, reliance on wild-collected material introduces additional challenges related to botanical identity, traceability, batch-to-batch phytochemical variability, environmental contamination, and harvesting sustainability. Commercial raw materials should therefore originate from controlled collection or cultivation systems and be supported by documented botanical authentication, traceability, contaminant control, and appropriate quality specifications, in accordance with Good Agricultural and Collection Practices (GACP) [122]. Potentially toxic elements have also been investigated in *A*. *ursinum* leaves. Ivanišová et al. reported Cd, Cr, and Pb only at trace levels, whereas Gordanić et al. found marked variation in potentially toxic element concentrations among samples collected from different locations in Serbia, with As, Cd, Cr, and Pb exceeding permitted levels at some sites [64,71].

*A*. *ursinum* is generally recognized as safe; however, potential adverse reactions associated with sulfur-containing compounds, allergenic constituents, and enhancement of anticoagulant therapy have been discussed [16]. No cases of hemolysis associated with Allium consumption have been reported in humans.

Nevertheless, important knowledge gaps remain. The currently available literature includes numerous studies investigating phytochemical composition and biological activities, including antioxidant, cytoprotective, cardioprotective, antiproliferative, antimicrobial, and antiparasitic effects [20,31,118,119,123,124]. By contrast, dedicated toxicological studies addressing acute toxicity, chronic exposure, reproductive toxicity, genotoxicity, mutagenicity, or long-term safety of concentrated *A*. *ursinum* preparations are scarce. Consequently, the current safety assessment of wild garlic relies primarily on its history of customary culinary use and the limited occurrence of reported intrinsic toxicity; this evidence should not be considered equivalent to comprehensive toxicological validation.

Overall, the available evidence is consistent with a history of customary culinary use of correctly identified *A*. *ursinum*, but dedicated toxicological studies addressing acute and chronic exposure, reproductive toxicity, genotoxicity, mutagenicity, and the long-term safety of concentrated preparations remain scarce. Consequently, the available evidence should not be considered equivalent to comprehensive toxicological validation or automatically extrapolated to highly concentrated extracts and supplements. Current toxicological concerns arise predominantly from accidental substitution with poisonous look-alike species, particularly *Colchicum autumnale*, *Convallaria majalis*, and *Arum maculatum*. Accurate botanical identification, authentication procedures, and quality-control measures throughout the food chain therefore remain essential for consumer safety.

Beyond these safety-related limitations, future research should prioritize the development of standardized preparations with well-defined phytochemical profiles and the establishment of dose-response relationships. Further studies are also needed to clarify the bioavailability and metabolism of key constituents, their potential synergistic or antagonistic interactions, and their behavior within food matrices. Ultimately, well-designed human intervention studies using standardized preparations and clinically relevant endpoints will be required before the biological activities observed experimentally can be translated into validated functional food applications.

## 8. Applications in Functional Food and Nutraceuticals

This section focuses on the incorporation of *A*. *ursinum* L. into food matrices for nutritional and functional enrichment, including effects on phytochemical content, antioxidant capacity, sensory properties, processing performance, formulation stability, and potential nutraceutical development.

The increasing interest in natural bioactive ingredients has led to the exploration of *A*. *ursinum* as a multifunctional component in food systems. Plant-derived compounds, particularly phenolics and flavonoids, are widely recognized for their antioxidant, antimicrobial, and preservative effects, which are essential for improving food quality and shelf life [98]. In parallel, the growing demand for clean-label foods and the need to replace synthetic additives such as nitrites have accelerated the use of plant-based ingredients in food formulations [99].

In this context, *A*. *ursinum* has emerged as a promising functional ingredient due to its rich phytochemical profile, including sulfur-containing compounds, phenolic acids, flavonoids, vitamins, and minerals. These constituents support the investigated use of *A*. *ursinum* in functional food formulations. By contrast, their application in nutraceutical preparations remains at an exploratory stage and requires further standardization, bioavailability assessment, and validation in human studies [27,125].

Building on the traditional and culinary uses discussed previously, the present section examines in greater detail the incorporation of *A*. *ursinum* into specific food matrices, processing strategies, and the technological and sensory outcomes reported in the literature. Potential nutraceutical applications are considered separately because the supporting evidence remains substantially more limited than that available for food products.

**Figure 8:**
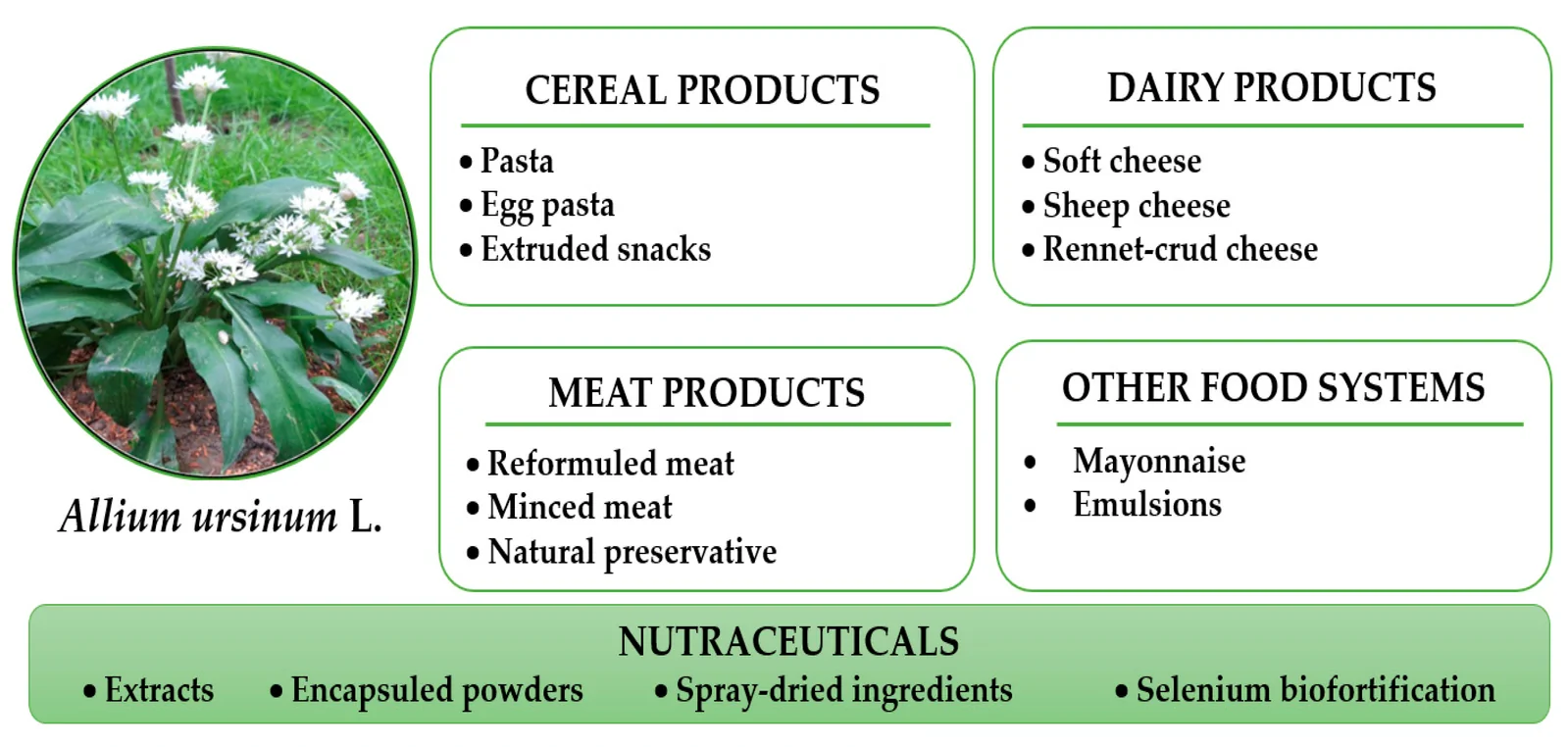
Documented food applications and potential formulation strategies for *Allium ursinum* L.

As illustrated in Figure 8, *A*. *ursinum* has been successfully incorporated into cereal, dairy, meat, and emulsified food products, whereas extracts and encapsulated preparations have mainly been investigated as potential ingredients for future functional food or nutraceutical formulations.

The documented food applications and investigated formulation strategies are summarized in Table 16.

### 8.1. Applications in Food Systems

The incorporation of *Allium ursinum* L. into diverse food matrices highlights its versatility as a functional ingredient. Among the investigated systems, cereal-based products-particularly pasta and extruded snacks-represent the most extensively studied applications, consistently demonstrating significant improvements in antioxidant capacity and phenolic content [27,29,58,59].

In pasta formulations, the form of incorporation plays a decisive role in determining both functional and technological performance. Powdered forms provide the most balanced outcome, ensuring enhanced bioactivity while maintaining acceptable sensory properties [27]. In contrast, the use of fresh leaves or blanching-derived ingredients may negatively affect cooking behavior and color parameters, indicating a trade-off between functionality and technological quality [58].

Dairy systems, particularly cheese, have also been explored as carriers of *A*. *ursinum*. While its addition improves antioxidant potential, it significantly alters the volatile profile and sensory characteristics, mainly due to sulfur-containing compounds [28,60,126].

In meat products, *A*. *ursinum* demonstrates promising potential as a natural preservative, contributing to enhanced oxidative stability, improved nutritional value, and extended shelf life [127,128]. Similar effects have been observed in emulsified systems such as mayonnaise, where the incorporation of extracts improves oxidative stability and microbiological safety [115].

Honey represents a distinct food matrix in which plant metabolites are transferred naturally rather than added during formulation. The volatile and semi-volatile profile of *A*. *ursinum* honey may support botanical-origin authentication, although the reported relative chromatographic signals should not be interpreted as concentrations in the original plant material [129].

Overall, these findings are consistent with broader research on plant-based additives, which emphasizes their capacity to enhance food quality, inhibit oxidation processes, and improve shelf life in complex food matrices [98].

### 8.2. Processing and Formulation Strategies

The functional performance of *Allium ursinum* L. is strongly influenced by processing techniques. Subcritical water extraction has been identified as an efficient method for recovering bioactive compounds, particularly phenolics and flavonoids [130]. However, the stability of these compounds remains a critical challenge.

Encapsulation techniques have been widely proposed as effective solutions for improving the stability, solubility, and potential delivery of plant-derived bioactives [131]. This approach is consistent with findings from other plant-based systems, where encapsulation enhances the stability of antioxidants and antimicrobial compounds, while reducing sensory drawbacks such as strong odor [98].

In addition, spray drying has been successfully applied to produce stable powdered extracts with high phenolic content and improved storage stability, supporting their potential use as standardized ingredients in functional foods or future nutraceutical formulations [125].

Overall, encapsulation, spray drying, and subcritical water extraction offer technological advantages in terms of the stability, solubility, and retention of bioactive compounds. However, these improvements should not be interpreted as evidence of increased gastrointestinal bioaccessibility, systemic bioavailability, or health efficacy unless these outcomes are demonstrated directly.

### 8.3. Functional and Nutraceutical Potential

In this review, functional foods refer to conventional food matrices formulated with *Allium ursinum* L. to provide technological or potential physiological benefits beyond basic nutrition, whereas nutraceuticals refer to concentrated preparations intended for use in non-conventional food forms, such as powders, capsules, or standardized extracts. The evidence supporting these two categories is currently unequal: several *A*. *ursinum*-enriched foods have been investigated experimentally, while specific nutraceutical formulations and their efficacy in humans remain insufficiently documented.

The growing interest in natural ingredients with health-promoting properties has increased attention toward *A*. *ursinum* as a potential component of functional foods and nutraceutical formulations. Its sulfur-containing compounds, phenolics, flavonoids, vitamins, minerals, and other phytochemicals support continued investigation of the species as a source of multifunctional ingredients [16].

The potential development of nutraceutical ingredients based on *A*. *ursinum* may benefit from advances in extraction and formulation technologies. Subcritical water extraction has been identified as an efficient and environmentally friendly approach for recovering phenolic compounds, flavonoids, and other bioactive constituents while avoiding the use of organic solvents [130]. Such extracts may serve as concentrated sources of phytochemicals for incorporation into functional foods and nutraceutical preparations.

To improve the stability, solubility, and potential delivery of these compounds, encapsulation technologies have been increasingly investigated. Tomšik et al. demonstrated that spray-congealing technology improved the stability, solubility, and antimicrobial activity of *A*. *ursinum* extracts, while Stupar et al. reported that spray drying with maltodextrin effectively preserved phenolics and flavonoids during storage. Evidence concerning gastrointestinal bioaccessibility remains limited. Kasprzak-Drozd et al. evaluated *A*. *ursinum*-enriched corn snacks using simulated gastric and duodenal digestion and reported substantial reductions in polyphenol and phenolic acid contents, as well as in antiradical activity, following digestion. Because this study employed an in vitro digestion model, its findings provide information on bioaccessibility but cannot establish systemic absorption or human bioavailability [29]. These approaches facilitate the production of standardized ingredients with enhanced shelf life and technological applicability.

In addition to extraction and encapsulation strategies, biofortification approaches have also been explored. Amagova et al. reported that selenium enrichment enhanced selenium accumulation, antioxidant status, and ascorbic acid content, increasing the potential of *A*. *ursinum* as a raw material for value-added functional foods.

Overall, current evidence supports the technological feasibility of using *A*. *ursinum* as an ingredient in several functional food matrices. Extracts, encapsulated preparations, and stabilized powders also indicate potential for future nutraceutical development; however, they should not be regarded as established nutraceutical products. Their further development requires compositional standardization, dose definition, bioavailability and pharmacokinetic studies, safety assessment, and confirmation of efficacy in well-designed human interventions.

### 8.4. Impact on Quality and Sensory Properties

Despite its numerous functional advantages, the incorporation of *Allium ursinum* L. may substantially influence the physicochemical, technological, and sensory characteristics of food products. The magnitude of these effects depends on the form of incorporation, concentration level, processing conditions, and the food matrix involved.

In cereal-based products, particularly pasta, the addition of *A*. *ursinum* has been associated with significant changes in color, texture, and cooking behavior. Rosan et al. reported that pasta enriched with fresh wild garlic leaves exhibited higher polyphenol content and antioxidant capacity while maintaining favorable sensory characteristics. However, increasing levels of wild garlic incorporation may affect technological parameters such as water absorption, swelling index, and cooking quality. Similarly, Rosan et al. observed reductions in lightness (L*) values, increased cooking losses, and noticeable color modifications in fortified pasta formulations, particularly when higher amounts of leaf powder or blanched leaves were used.

Comparable observations were reported by Filipčev et al., who demonstrated that different wild garlic preparations significantly increased the antioxidant potential of pasta products, although excessive enrichment levels negatively affected certain cooking and textural properties. Among the tested formulations, powder-based incorporation provided the most favorable balance between functional enhancement and sensory acceptance.

In dairy products, *A*. *ursinum* markedly influences aroma and flavor characteristics due to the presence of sulfur-containing volatile compounds. Pluta-Kubica et al. demonstrated that the addition of wild garlic leaves altered the volatile profile of soft cow milk cheese, resulting in less pronounced milky and sour notes while maintaining overall sensory acceptability. Similar results were observed in sheep milk cheese, where wild garlic enhanced herbal aroma and piquant taste without negatively affecting overall product quality [60]. Furthermore, bear garlic supplementation increased antioxidant capacity in soft rennet cheeses while preserving microbiological quality and product stability [126].

In meat products, the characteristic sulfur-derived flavor of *A*. *ursinum* may contribute positively to product acceptability while simultaneously enhancing oxidative stability and shelf life [127,128]. Likewise, the incorporation of wild garlic into mayonnaise formulations improved both antioxidant and microbiological stability without compromising product quality [115].

Overall, the optimal concentration of *A*. *ursinum* is likely to be product-specific. Increasing the amount incorporated may enhance phenolic content and antioxidant potential while simultaneously affecting color, texture, cooking properties, flavor, or consumer acceptance. Formulation levels should therefore be optimized separately for each food matrix to balance functional enhancement with technological and sensory quality.

### 8.5. Critical Considerations and Future Perspectives

Although *Allium ursinum* L. demonstrates considerable potential as a functional food ingredient and as a possible source of future nutraceutical preparations, several limitations continue to restrict its broader industrial application. One of the main challenges is the considerable phytochemical variability associated with biological source and harvest conditions, which may affect the functional performance of food products [41,70,87]. Reducing inter-study and batch-to-batch variability will require better standardization of plant material, harvest conditions, extraction procedures, analytical methods, and reporting formats. Because functional effects also depend on preparation, concentration, and processing conditions, reproducible specifications for key sulfur-containing compounds and phenolics will be necessary to translate experimental findings into consistent commercial products. Another important limitation is related to the strong sulfur-derived aroma and flavor characteristic of *A*. *ursinum*. While these sensory attributes may be desirable in certain food categories, excessive concentrations can negatively influence consumer acceptance, particularly in products with mild flavor profiles. Therefore, optimization of formulation strategies and dosage levels remains essential for successful product development.

The stability of bioactive compounds during processing and storage also represents a critical issue. Sulfur compounds, phenolics, and other phytochemicals may undergo degradation during thermal treatments, drying, storage, or exposure to oxygen. Consequently, advanced technologies such as subcritical water extraction, spray drying, encapsulation, and controlled-release systems have attracted increasing attention as strategies to improve the preservation and delivery of bioactive constituents [125,130,131].

Despite promising laboratory-scale results, most currently available studies have been conducted under controlled experimental conditions. Information regarding industrial-scale processing, long-term storage stability, bioavailability, consumer acceptance, and economic feasibility remains relatively limited. Food-matrix studies are available but predominantly address technological, physicochemical, antioxidant, antimicrobial, and sensory outcomes rather than health effects in consumers. Importantly, evidence obtained in food matrices primarily demonstrates technological or compositional performance and should not be considered equivalent to evidence of physiological efficacy in consumers. Furthermore, clinical evidence supporting the health benefits of *A*. *ursinum*-enriched foods remains scarce, highlighting the need for additional human intervention studies [23,26]. More specifically, no controlled human efficacy trials or human pharmacokinetic studies were identified, while the available digestion data are limited to in vitro bioaccessibility rather than systemic bioavailability [29].

Future research should focus on controlled cultivation and processing protocols, optimization of extraction and encapsulation technologies, evaluation of bioavailability and efficacy in vivo, and the design of innovative food products with improved sensory acceptance. Although growing consumer demand for clean-label products, natural preservatives, and sustainable food systems provides a favorable context, the commercial feasibility of *A*. *ursinum*-based products remains to be established through industrial-scale validation, economic assessment, product standardization, and consumer studies [99].

Overall, the available evidence supports *A*. *ursinum* as a promising candidate for functional food development, potential nutraceutical formulations, and natural food-preservation systems. However, its broader commercial application requires reproducible production systems, industrial-scale processing, long-term stability assessment, safety and efficacy validation, and well-designed human intervention studies.

The broader technological implications of these properties, particularly the potential use of *A*. *ursinum* as a natural alternative to conventional food additives, are discussed separately in the following section.

## 9. Wild Garlic as a Potential Clean-Label Technological Ingredient

Whereas the preceding section focuses on the incorporation of *Allium ursinum* L. into food products for nutritional and functional enrichment, the present section specifically examines its potential technological role as a clean-label ingredient capable of performing functions conventionally associated with food additives. These include antioxidant protection, microbial control, flavor contribution, and, to a lesser extent, color-related effects.

The suitability of *A*. *ursinum* for these applications is primarily associated with its combination of sulfur-containing compounds, phenolics, flavonoids, chlorophylls, and carotenoids [16,43]. These constituents may contribute antioxidant, antimicrobial, sensory, and technological functions and could contribute to reducing reliance on selected synthetic additives in suitable food matrices.

**Figure 9:**
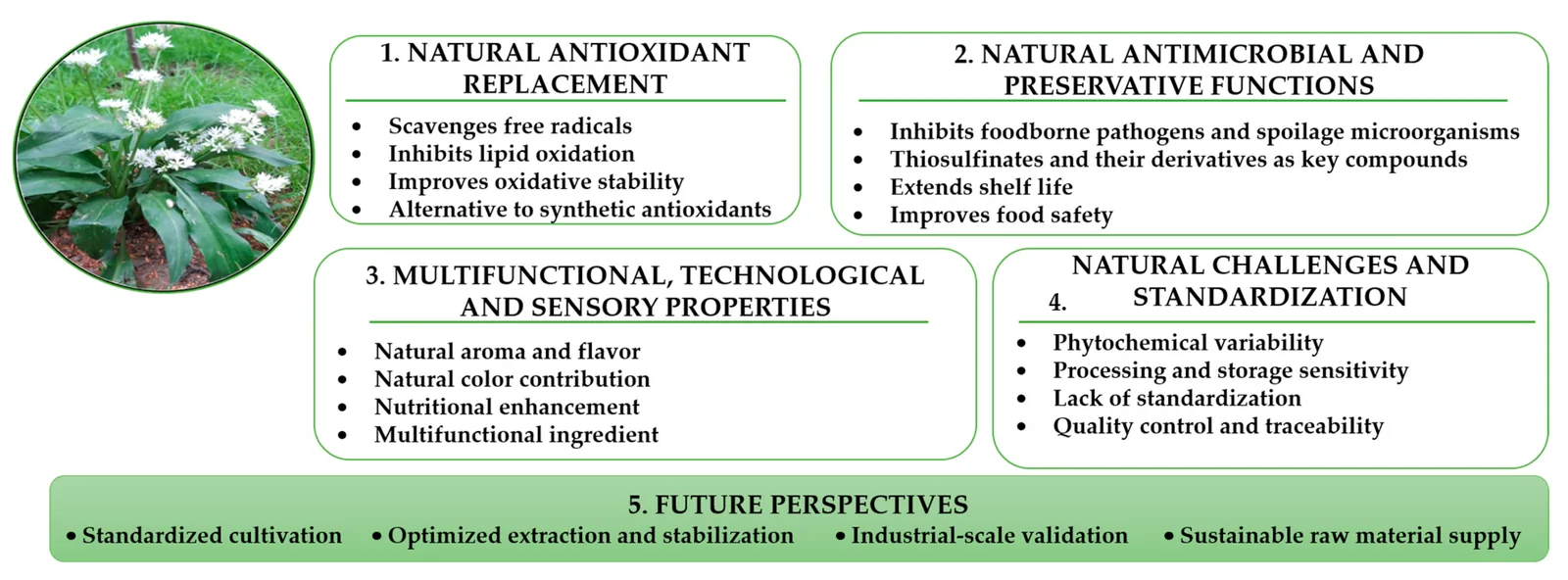
Conceptual framework for the potential use of *Allium ursinum* L. as a multifunctional clean-label ingredient in food systems.

However, most available evidence derives from laboratory assays, food-model systems, or small-scale product studies. Therefore, the current evidence supports the potential use of *A*. *ursinum* as a multifunctional clean-label ingredient rather than its established replacement of conventional additives in commercial food production. The main technological roles and limitations are summarized in Figure 9.

### 9.1. Potential Natural Antioxidant Function

One potential clean-label application of *A*. *ursinum* L. is its use as a natural antioxidant ingredient to help control oxidative deterioration in food systems. The antioxidant activity of wild garlic has been attributed mainly to its phenolic compounds, flavonoids, sulfur-containing metabolites, and antioxidant enzymes [16,100].

Several studies have demonstrated that *A*. *ursinum* extracts effectively inhibit oxidative processes in food matrices. Pressurized-liquid extracts exhibited strong antioxidant activity and improved oxidative stability in lipid systems [103]. Similar effects were observed in mayonnaise formulations, where the incorporation of wild garlic significantly increased antioxidant capacity and contributed to product stabilization during storage [115]. In meat products, wild garlic supplementation improved oxidative stability and enhanced overall product quality, supporting its potential as a natural antioxidant ingredient [127].

Although promising, the antioxidant performance of *A*. *ursinum* varies with plant material, extraction procedure, food matrix, and processing conditions. Further standardization and direct comparison with conventional antioxidants are therefore required before its technological suitability as an alternative antioxidant ingredient can be established [83].

### 9.2. Natural Antimicrobial and Preservative Functions

In addition to its antioxidant properties, *Allium ursinum* L. has demonstrated antimicrobial activity under experimental conditions, supporting its investigation as a potential natural preservative. Sulfur-containing compounds, particularly thiosulfinates and their degradation products, have been identified as the main contributors to antimicrobial effectiveness [16].

The antimicrobial activity of *A*. *ursinum* extracts has been reported against a broad spectrum of microorganisms, including foodborne pathogens and spoilage organisms [113]. Moreover, extracts produced through green extraction technologies demonstrated inhibitory activity against *Listeria monocytogenes* and other relevant foodborne bacteria, supporting their potential application in food preservation systems [109]. Pressurized-liquid extracts also exhibited antimicrobial activity while simultaneously contributing to oxidative stability [103].

However, most of the available antimicrobial evidence has been obtained using in vitro assays or isolated experimental systems [103,125]. Activity demonstrated under these conditions should be distinguished from preservation efficacy in complex food matrices, where fat, protein, water activity, pH, processing conditions, and interactions with other ingredients may reduce or modify antimicrobial performance [133]. Consequently, direct evidence of microbial control and shelf-life extension in specific food matrices is required before its use as an alternative preservative can be considered technologically validated.

The combination of antioxidant and antimicrobial functions represents a potential advantage because a single ingredient could contribute to oxidative protection and microbial control. Nevertheless, its contribution to shelf-life extension and food safety must be confirmed through challenge tests and storage studies conducted in representative food matrices [133].

### 9.3. Multifunctional Technological and Sensory Properties

Beyond antioxidant and antimicrobial functions, *Allium ursinum* L. may also serve as a natural flavoring ingredient because of its characteristic sulfur-derived aroma. This sensory contribution is technologically relevant in products where garlic-like, herbal, or pungent notes are desirable, including meat, cheese, sauces, and selected cereal-based products [43]. However, this function is strongly dose- and matrix-dependent. At excessive incorporation levels, the characteristic sulfurous aroma and flavor may dominate the sensory profile and reduce consumer acceptance, particularly in foods with mild or neutral flavor characteristics. Therefore, its use as a natural flavoring ingredient requires matrix-specific optimization rather than generalized substitution of conventional flavorings.

Studies have shown that the concentration and composition of volatile sulfur compounds vary according to harvest stage, with higher concentrations generally observed before flowering [6]. These multifunctional properties may reduce the need for additional flavoring or coloring agents in certain food products. For example, wild garlic modified the volatile profile and sensory attributes of cheeses [28,60], while in pasta products it increased phenolic content and antioxidant activity, although changes in color and cooking behavior were also observed [27,58].

These effects are concentration-dependent and must be evaluated for each food matrix. The characteristic sulfur-containing aroma and flavor may be advantageous in products such as meat or cheese, where garlic-like sensory notes are compatible with the expected product profile. However, excessive incorporation levels may produce an overpowering aroma or flavor and reduce consumer acceptance, particularly in foods with a neutral or delicate sensory profile. Increasing the amount of *A*. *ursinum* may also improve phenolic content and antioxidant potential while simultaneously affecting color, texture, cooking properties, and overall acceptability. Therefore, optimal incorporation levels should be established separately for each product through technological measurements and sensory and consumer evaluation [24,25,64,66].

### 9.4. Industrial Challenges and Standardization

Despite its promising potential, several challenges continue to limit the industrial implementation of *A*. *ursinum* L. as a clean-label ingredient. A major industrial challenge is batch-to-batch variability in phytochemical composition, which may compromise reproducible technological performance [6,9,70].

Furthermore, sulfur-containing compounds are highly sensitive to temperature, oxygen exposure, and storage conditions, which may result in losses of bioactivity and alterations in sensory properties [125,131]. Maintaining consistent quality and functionality therefore requires well-controlled processing and storage conditions.

Another challenge involves the absence of standardized specifications for *A*. *ursinum*-derived ingredients. Variability in extract composition may affect reproducibility and complicate the technological use of A. *ursinum*-derived ingredients as potential alternatives to conventional food additives. This lack of standardization is closely linked to difficulties in quality assurance and authentication of raw materials.

Additional challenges are related to quality control and authenticity. Because *A*. *ursinum* is frequently collected from natural habitats, the risks of species misidentification, adulteration, and inconsistencies in raw material quality remain significant. Reliable authentication methods, traceability systems, and standardized quality specifications are therefore essential to ensure product safety, consistency, and consumer confidence [121].

### 9.5. Future Perspectives for Clean-Label Applications

Future research should focus on the development of standardized cultivation, extraction, and stabilization protocols capable of ensuring consistent phytochemical composition and technological performance. Additional studies are also required to evaluate the efficacy of *A*. *ursinum* L.-derived ingredients under industrial processing conditions and during long-term storage [125,130,131].

The availability of raw material may also become a limiting factor for future industrial development. *A*. *ursinum* is harvested during a relatively short seasonal period, and, in some European countries, wild populations are protected by legal regulations. Moreover, intensive harvesting from natural habitats may place additional pressure on local populations and cannot ensure the uniformity of raw materials required for food-industry applications. Therefore, the development of controlled cultivation systems should be prioritized over reliance on large-scale wild harvesting. Cultivation under defined environmental and agronomic conditions could support a more reliable and traceable supply while facilitating greater consistency in phytochemical composition, safety, and technological performance [6,9,36,51,70,132]. Sustainable harvesting practices may remain appropriate for limited local use, but commercial-scale applications will require responsible sourcing, cultivation protocols, and quality specifications adapted to *A*. *ursinum*.

The growing demand for natural preservatives, clean-label products, and sustainable food systems is expected to further increase interest in *A*. *ursinum* [98,99]. The reported antioxidant, antimicrobial, sensory, and compositional properties support further investigation of *A*. *ursinum* as a multifunctional clean-label technological ingredient, although product-specific validation and comparison with conventional additives remain necessary.

## 10. Conclusions

This review consolidates current knowledge on *Allium ursinum* L. and supports its potential as a multifunctional ingredient for functional foods and clean-label formulations. Its complex phytochemical profile includes sulfur-containing compounds, polyphenols, vitamins, minerals, pigments, and other specialized constituents that contribute to its nutritional, sensory, antioxidant, antimicrobial, and technological properties. However, substantial compositional variability remains a major challenge for standardization.

The available evidence supports antioxidant, antimicrobial, cardiovascular, and cytoprotective or redox-modulating activities under specific experimental conditions. Nevertheless, most evidence supporting the reported biological activities originates from chemical assays, cell-based experiments, or animal models, whereas food-matrix studies primarily address technological performance and controlled human efficacy studies remain lacking. Consequently, the reported biological activities should be interpreted as evidence of functional potential rather than as confirmation of clinical efficacy or substantiated human health benefits.

From a technological perspective, *A*. *ursinum* has been successfully incorporated into cereal, dairy, meat, and emulsified food products, where it may improve phytochemical content, oxidative stability, sensory characteristics, and, in some cases, microbiological quality. However, these effects depend on the food matrix, the form of the ingredient, and its concentration. The characteristic sulfur-derived aroma and flavor may enhance products compatible with garlic-like sensory notes but may limit acceptance in foods with neutral or delicate sensory profiles. Future product development should therefore establish matrix-specific incorporation levels and evaluate consumer acceptance, repeated-consumption acceptability, and sensory changes during storage alongside technological performance.

Reliable industrial application will require standardized and authenticated raw materials, defined cultivation and harvesting conditions, marker-based phytochemical specifications, and validated processing and storage protocols. Further research should also address gastrointestinal bioaccessibility, human bioavailability, dose-response relationships, and the safety of concentrated extracts and supplements. Botanical authentication, traceability, contaminant control, and regulatory requirements corresponding to the intended product category and jurisdiction should be integrated into future development strategies. Controlled cultivation and responsible sourcing will also be essential for ensuring a consistent supply while limiting pressure on wild populations.

In conclusion, *A*. *ursinum* represents a promising, multifunctional, and potentially sustainable clean-label ingredient. Nevertheless, its transition from an underexploited wild edible plant to standardized functional food and nutraceutical applications will depend on reproducible raw materials, validated processing technologies, stronger human evidence, clearer safety and regulatory frameworks, demonstrated bioavailability, and product-specific confirmation of consumer acceptance.

## Figures and Tables

**Table 1:** Taxonomic classification of *Allium ursinum* subsp. *ursinum* according to the Catalogue of Life [8].

Taxonomic Rank	Classification
Domain	Eukaryota
Kingdom	Plantae
Subkingdom	Pteridobiotina
Phylum	Tracheophyta
Class	Liliopsida
Order	Asparagales
Family	Amaryllidaceae
Genus	*Allium*
Species	*Allium ursinum* L.
Subspecies	*Allium ursinum* subsp. *ursinum*

**Table 2:** Proximate composition of *Allium ursinum* L. reported in different studies.

Parameter	Plant Part	Sample Condition	Quantity	Unit	Method	Reference
Dry matter	Leaves	Fresh	79.0 ± 4.1	g kg^−1^ FW	oven drying	[65]
	Leaves	Fresh	10.73 ± 0.25	% FW	oven drying	[66]
	Leaves	Fresh *	9.79–10.77	% FW	oven drying	[63]
	Leaves	Fresh	14.9 ± 0.06	% FW	oven drying	[67]
	Stems	Fresh *	9.61–11.26	% FW	oven drying	[63]
	Bulbs	Fresh *	26.74–27.79	% FW	oven drying	[63]
Protein (crude)	Leaves	Fresh	13.7 ± 0.2	g kg^−1^ FW	AOAC 950.36	[65]
	Leaves	Fresh	4.03 ± 0.05	% FW	Kjeldahl	[66]
	Leaves	Fresh *	19.33–26.53	g 100 g^−1^ DW	AOAC 950.36	[63]
	Leaves	Frozen	4.0	g 100 g^−1^	PB-116, ed. III	[68]
	Stems	Fresh *	6.23–16.12	g 100 g^−1^ DW	AOAC 950.36;	[63]
	Bulbs	Fresh *	19.83–20.62	g 100 g^−1^ DW	AOAC 950.36	[63]
Fat	Leaves	Fresh	5.6 ± 0.0	g kg^−1^ FW	AOAC 935.38	[65]
	Leaves	Fresh *	2.32–4.98	g 100 g^−1^ DW	AOAC 935.38	[63]
	Leaves	Frozen	0.6	g 100 g^−1^	PN-A-82100:1985	[68]
	Stems	Fresh *	0.52–1.87	g 100 g^−1^ DW	AOAC 935.38	[63]
	Bulbs	Fresh *	0.47–1.12	g 100 g^−1^ DW	AOAC 935.38	[63]
Total soluble sugars	Leaves	Fresh	1.34 ± 0.12	% FW	Somogyi-Nelson	[66]
Total sugar	Leaves	Fresh	5.7 ± 0.17	% FW	Bertrand	[67]
Reducing sugars	Leaves	Fresh; water extract	~2.7	g glucose equivalents 100 g^−1^ extract	DNS	[69]
Total carbohydrates	Leaves	Fresh	50.8 ± 3.6	g kg^−1^ FW	Calculated by difference	[65]
	Leaves	Fresh *	1.74–2.99	g 100 g^−1^ DW	Anthrone colorimetric method	[63]
	Leaves	Fresh; water extract *	~5.3	g glucose equivalents 100 g^−1^ extract	Phenol-sulfuric acid spectrophotometric assay	[69]
	Stems	Fresh *	4.07–5.09	g 100 g^−1^ DW	Anthrone colorimetric method	[63]
	Bulbs	Fresh *	0.96–2.73	g 100 g^−1^ DW	Anthrone colorimetric method	[63]
Dietary fiber	Leaves	Fresh	26.9 ± 0.5	g kg^−1^ FW	AOAC 991.43	[65]
	Leaves	Frozen	2.5	g·100 g^−1^FW	AOAC 991.43	[68]
Ash	Leaves	Fresh	8.9 ± 0.1	g kg^−1^ FW	AOAC 930.05	[65]
	Leaves	Fresh *	7.69–7.78	g 100 g^−1^ DW	AOAC 930.05	[63]
	Stems	Fresh *	6.36–7.76	g 100 g^−1^ DW	AOAC 930.05	[63]
	Bulbs	Fresh *	2.72–4.11	g 100 g^−1^ DW	AOAC 930.05	[63]

Notes: FW, fresh-weight basis; DW, dry-weight basis. Dry matter represents the fraction remaining after water removal, whereas ash represents the inorganic residue remaining after incineration. Values expressed on an FW basis should not be directly compared with those expressed on a DW basis without appropriate conversion. * Values represent the minimum and maximum contents determined in samples harvested in 2015 and 2016.

**Table 3:** Vitamin C composition of *Allium ursinum* L.

Compound	Plant/Part	Condition/Stage	Quantity	Unit	Method	Reference
Vitamin C	Leaves	Fresh *	97.0 ± 1.15	mg 100 g^−1^ FW	2,6-dichlophenolindophenol titration	[67]
	Leaves	Fresh *	24.20–49.70	mg 100 g^−1^ FW	Tillmans titration	[63]
	Stems	Fresh *	18.47–41.14	mg 100 g^−1^ FW	Tillmans titration	[63]
	Bulbs	Fresh *	5.43–6.11	mg 100 g^−1^ FW	Tillmans titration	[63]

* Values represent the minimum and maximum contents determined in samples harvested in 2015 and 2016.

**Table 4:** Mineral composition of *Allium ursinum* L. reported in different studies.

Mineral	Plant/Part	Quantity	Unit	Method	Reference
**Potassium (K)**	Leaves	34.64 ± 1.21	g kg^−1^ DW	dry ashing + ICP-AES	[65]
	Leaves	10,765.8	mg kg^−1^ FW	AAS	[63]
	Leaves	2655 ± 10.81	mg 100 g^−1^	ICP-OES	[64]
	Leaves	3291.25 ± 87.12	mg kg^−1^ FW	ICP-OES	[71]
**Calcium (Ca)**	Leaves	13.44 ± 0.02	g kg^−1^ DW	dry ashing + ICP-AES	[65]
	Leaves	338.6	mg kg^−1^ FW	AAS	[63]
	Leaves	702 ± 2.59	mg 100 g^−1^	ICP-OES	[64]
	Leaves	324.35 ± 16.87	mg kg^−1^ FW	ICP-OES	[71]
**Magnesium (Mg)**	Leaves	1.72 ± 0.03	g kg^−1^ DW	dry ashing + ICP-AES	[65]
	Leaves	470.7	mg kg^−1^ FW	AAS	[63]
	Leaves	251 ± 3.75	mg 100 g^−1^	ICP-OES	[64]
	Leaves	281.85 ± 11.52	mg kg^−1^ FW	ICP-OES	[71]
**Phosphorus (P)**	Leaves	3578.5	mg kg^−1^ FW	Spectrophotometry	[63]
	Leaves	391 ± 2.13	mg 100 g^−1^	ICP-OES	[64]
**Sulfur (S)**	Leaves	5.08 ± 0.03	g kg^−1^ DW	dry ashing + ICP-AES	[65]
	Leaves	932 ± 3.21	mg 100 g^−1^	ICP-OES	[64]
**Iron (Fe)**	Leaves	230.34 ± 9.55	mg kg^−1^ DW	dry ashing + ICP-AES	[65]
	Leaves	9.1	mg kg^−1^ FW	AAS	[63]
	Leaves	12.3 ± 1.24	mg 100 g^−1^	ICP-OES	[64]
	Leaves	53.58 ± 8.15	mg kg^−1^ FW	ICP-OES	[71]
**Zinc (Zn)**	Leaves	58.76 ± 7.14	mg kg^−1^ DW	dry ashing + ICP-AES	[65]
	Leaves	21.2	mg kg^−1^ FW	AAS	[63]
	Leaves	1.34 ± 0.08	mg 100 g^−1^	ICP-OES	[64]
	Leaves	10.06 ± 2.57	mg kg^−1^ FW	ICP-OES	[71]
**Copper (Cu)**	Leaves	0.46 ± 0.11	mg 100 g^−1^	ICP-OES	[64]
	Leaves	1.53 ± 0.22	mg kg^−1^ FW	ICP-OES	[71]
**Manganese (Mn)**	Leaves	3.0	mg kg^−1^ FW	AAS	[63]
	Leaves	6.00 ± 1.98	mg 100 g^−1^	ICP-OES	[64]
	Leaves	4.99 ± 2.98	mg kg^−1^ FW	ICP-OES	[71]
**Sodium (Na)**	Leaves	84.3	mg kg^−1^ FW	AAS	[63]
	Leaves	3.24 ± 1.11	mg 100 g^−1^	ICP-OES	[64]
	Leaves	20.94 ± 8.66	mg kg^−1^ FW	ICP-OES	[71]

Note: FW, fresh-weight basis; DW, dry-weight basis. Values are reported according to the expression basis specified in the original studies and should not be directly compared without appropriate conversion. The reporting basis (FW/DW) for mineral values reported by Ivanišová et al. was not specified in the original article. Values reported by Gordanić et al. represent mean concentrations ± SD obtained from *A. ursinum* L. leaf samples collected across 43 locations in Serbia.

**Table 5:** Chemical structures of representative phytochemicals reported in *Allium ursinum* L.

A. Sulfur-Containing Compounds			B. Representative Flavonoids
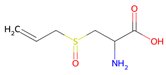	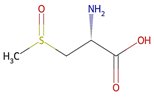	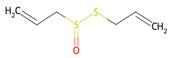	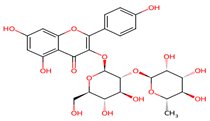
Alliin [74] C_6_H_11_NO_3_S	Methiin [75] C_4_H_9_NO_3_S	Allicin [76] C_6_H_10_OS_2_	Kaempferol 3-O-β neohesperidoside [77] C_27_H_30_O_15_
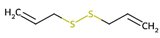	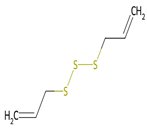	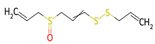	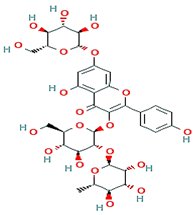
Diallyl disulfide [78] C_6_H_10_S_2_	Diallyl trisulfide [79] C_6_H_10_S_3_	Ajoene [80] C_9_H_14_OS_3_	Kaempferol 3-O-β-neohesperidoside-7-O-β-D-glucopyranoside [81] C_33_H_40_O_20_

Notes: Chemical structures and molecular formulas were obtained from ChemSpider and PubChem; the corresponding database records are cited in the table.

**Table 6:** Sulfur-containing compounds identified in *Allium ursinum* L. and their methodological context.

Compound	Chemical Class	Plant Part	Condition/Stage	Quantity	Analytical Method	Reference
Methiin (S-methyl-L-cysteine sulfoxide)	ACSO precursor	Leaves	Fresh	60.0 mg/100 g FW	GC-FID after ethyl chloroformate derivatization and NaI reduction	[7]
	ACSO precursor	Bulbs Leaves	n.s.	62% of total ACSOs 74% of total ACSOs	HPLC after PITC derivatization	[17]
	ACSO precursor	Bulbs Leaves		0.80% DW 0.40% DW	HPLC after OPA derivatization	[15]
Ethiin (S-ethyl-L-cysteine sulfoxide)	Minor ACSO precursor	Leaves	Fresh	0.4 mg/100 g FW	GC-FID after ethyl chloroformate derivatization and NaI reduction	[7]
Propiin (S-propyl-L-cysteine sulfoxide)	ACSO precursor	Leaves	Fresh	1.2 mg/100 g FW	GC-FID after ethyl chloroformate derivatization and NaI reduction	[7]
Alliin (S-allyl-L-cysteine sulfoxide)	ACSO precursor	Leaves	Fresh	40.3 mg/100 g FW	GC-FID after ethyl chloroformate derivatization and NaI reduction	[7]
	ACSO precursor	Bulbs Leaves	n.s.	38% of total ACSOs 26% of total ACSOs	HPLC after PITC derivatization	[17]
	ACSO precursor	Leave Bulbs	Dried Dried	0.20% DW 0.75% DW	HPLC after OPA derivatization	[15]
Isoalliin (S-(E)-1-propenyl-L-cysteine sulfoxide)	ACSO precursor	Leaves	Fresh	trace < 0.2 mg/100 g FW	GC-FID after ethyl chloroformate derivatization and NaI reduction	[7]
Allicin (diallyl thiosulfinate)	Thiosulfinate	Leaves Bulbs	n.s.	0.13% DW 0.53% DW	HPLC of chloroform extract from freshly disrupted material	[15]
Allyl-methyl/methyl-allyl thiosulfinate (MATS)	Mixed thiosulfinate	Leaves Bulbs	n.s.	0.26% DW 0.70% DW	HPLC of chloroform extract from freshly disrupted material	[15]
Dimethyl thiosulfinate	Thiosulfinate	Leaves Bulbs	n.s.	0.13% DW 0.27% DW	HPLC of chloroform extract from freshly disrupted material	[15]
Thiosulfinate profile in freeze-dried wild garlic extract (total 21 µmol/g)	Molar composition	n.s.	Freeze-dried powder extract	Allicin 28 mol%; AllS(O)SMe 16 mol%; AllSS(O)Me 34 mol%; MeS(O)SMe 20 mol%; AllS(O)SPropenyl-(Z,E) 0.9 mol%; MeS(O)SPropenyl-(Z,E) 0.7 mol%	Si-HPLC	[5]
Allyl methyl disulfide (methyl 2-propenyl disulfide) (AMDS)	Volatile disulfide	Leaves	Volatile oil	5.07%	Hydrodistillation + GC-MS/GC-FID	[83]
		Aerial parts/inflorescences	Volatile oil	13.0–18.9% (A-E); 1.1% (F); 8.6% (G); 10.0–12.8% (H)	Hydrodistillation + GC-MS/GC-FID	[82]
Methyl (E)-1-propenyl disulfide	Volatile disulfide	Aerial parts/inflorescences	Volatile oil	3.4–6.2% (A-E); 0.3% (F); 0.9% (G); 2.4–3.0% (H)	Hydrodistillation + GC-MS/GC-FID	[82]
Allyl (E)-1-propenyl disulfide	Volatile disulfide	Aerial parts/inflorescences	Volatile oil	7.5–10.2% (A-E); 4.4% (F); 3.4% (G); 6.2–8.4% (H)	Hydrodistillation + GC-MS/GC-FID	[82]
Diallyl disulfide (di-2-propenyl disulfide) (DADS)	Volatile disulfide	Fresh aerial parts	Volatile oil	16.2–21.2% (A-E); 9.9% (F); 10.0% (G); 18.2–20.7% (H)	Hydrodistillation + GC-MS/GC-FID	[82]
Dimethyl trisulfide (DMTS)	Volatile trisulfide	Aerial parts/inflorescences	Volatile oil	3.5–7.5% (A-E); 1.1% (F); 7.0% (G); 4.2–5.5% (H)	Hydrodistillation + GC-MS/GC-FID	[82]
Allyl methyl trisulfide (methyl 2-propenyl trisulfide) (AMTS)	Volatile trisulfide	Leaves	Volatile oil	14.16%	Hydrodistillation + GC-MS/GC-FID	[83]
		Aerial parts/inflorescences	Volatile oil	12.6–15.0% (A-E); 11.4% (F); 26.5% (G); 15.2–17.1% (H)	Hydrodistillation + GC-MS/GC-FID	[82]
Diallyl trisulfide (di-2-propenyl trisulfide) (DATS)	Volatile trisulfide	Fresh aerial parts	Volatile oil	4.9–8.7% (A-E); 13.2% (F); 19.6% (G); 11.4–12.5% (H)	Hydrodistillation + GC-MS/GC-FID	[82]
Dimethyl disulfide (DMDS)	Volatile disulfide	Aerial parts/inflorescences	Volatile oil	0.7–2.3% (A-E); tr (F); 0.8% (G); 0.7–0.8% (H)	Hydrodistillation + GC-MS/GC-FID	[82]
Ajoene	Sulfur rearrangement products	Bulb	Extracts	identified	HPLC/MPLC; confirmation by NMR and MS	[15]
Methylajoene, dimethylajoene	Sulfur rearrangement products	Bulb	Chloroform extract	identified	MPLC; identification by UV, MS and NMR	[15]
2-vinyl-4H-1,3-dithiin	Cyclic sulfur compound	Bulb	Extracts	identified	HPLC	[15]
3-vinyl-,2-dithiin	Cyclic sulfur compound	Bulb	Extracts	identified	HPLC	[15]

Notes. n.s., -not specified; ACSO, S-alk(en)yl-L-cysteine sulfoxide; FW, fresh weight; DW, dry weight; DADS, diallyl disulfide; DATS, diallyl trisulfide; AMDS, allyl methyl disulfide; AMTS, allyl methyl trisulfide; DMDS, dimethyl disulfide; DMTS, dimethyl trisulfide; GC-MS, gas chromatography-mass spectrometry; GC-FID, gas chromatography-flame ionization detection; HPLC, high-performance liquid chromatography; MPLC, medium-pressure liquid chromatography; PITC, phenyl isothiocyanate; OPA, o-phthalaldehyde. Values reported by Kubec et al. and Sendl and Wagner are expressed on FW and DW bases, respectively. Thiosulfinate values reported by Block et al. expressed as molar percentages of the generated thiosulfinate fraction, whereas volatile-compound percentages reported by Gođevac et al. and Radulović et al. represent relative peak-area percentages in essential oils. In the latter study, A-E denote fresh aerial samples, F air-dried material, G oven-dried material, and H fresh inflorescences. “Identified” indicates qualitative detection without reliable quantification. Because the data differ in plant material, processing, analytical method, and reporting basis, direct quantitative comparisons among studies are not appropriate.

**Table 7:** (**A**) Total phenolic content of *Allium ursinum* L. according to plant organ and extraction method. (**B**) Major individual phenolic compounds and chromatographic phenolic profiles reported in *Allium ursinum* L.

(**A**)						
**Compound/Indicator**	**Plant Organ**	**Concentration/Unit**		**Extraction/Analytical Method**		**Reference**
Total phenolic content/Polyphenolic pool	Leaves	Free fraction: 3.24 ± 0.29 mg FAE/g DW; Bound fraction: 1.10 ± 0.14 mg FAE/g DW; Total: 4.34 mg FAE/g DW		80% boiling aqueous methanol + ethyl acetate extraction; Folin-Ciocalteu assay; acid hydrolysis of residue for bound fraction		[85]
	Bulbs	Free fraction: 2.30 ± 0.11 mg FAE/g DW; Bound fraction: 1.00 ± 0.11 mg FAE/g DW; Total: 3.30 mg FAE/g DW		80% boiling aqueous methanol + ethyl acetate extraction; Folin-Ciocalteu assay; acid hydrolysis of residue for bound fraction		[85]
	Leaves	April: 726 ± 10.1 mg GAE/kg FW;		80% methanol extraction; Folin-Ciocalteu assay		[87]
		May: 782 ± 11.2 mg GAE/kg FW;				
		June: 827 ± 9.82 mg GAE/kg FW;				
	Flowers	May: 1410 ± 12.5 mg GAE/kg FW				
	Leaves Flowers Bulbs	642.95–1069.51 mg GAE/kg FW 435.05–668.21 mg GAE/kg FW 253.19–283.51 mg GAE/kg FW		80% methanol extraction; Folin-Ciocalteu assay		[88]
	Leaves	242.04 ± 10.46 mg GAE/100 g FW		Modified Folin-Ciocalteu assay		[66]
	Leaves	132.15–186.18 mg GAE/100 g FW		80% EtOH extraction; Folin-Ciocalteu assay		[70]
	Bulbs Leaves	11.2 ± 0.3 mg GAE/g DW; 10.4 ± 0.2 mg GAE/g DW		80:20 methanol/water extraction; ultrasonic bath; Folin-Ciocalteu assay		[84]
(**B**)						
**Compound/Indicator**	**Class**	**Plant** **Organ**	**Concentration/Unit**	**Phenolic** **Fraction**	**Extraction/Analytical Method**	**Reference**
Gallic acid	Phenolic acid	Leaves	75.8–322.5 mg/100 g	n.s.	Methanolic extraction; HPLC-DAD	[13]
*p*-Coumaric acid	Phenolic acid	Leaves	trace 25.43 ± 1.45 µg/g DW	Free Bound	80% boiling aqueous MeOH + EtOAc; acid hydrolysis; HPLC-DAD	[85]
	Phenolic acid	Bulbs	trace 37.38 ± 2.87 µg/g DW	Free Bound	80% boiling aqueous MeOH + EtOAc; acid hydrolysis; HPLC-DAD	[85]
	Phenolic acid	Leaves	109.11 µg/100 g	non-hydrolyzed	70% ethanol, repercolation; HPLC-UV-MS; acid hydrolysis	[89]
			369.03 µg/100 g	hydrolyzed		
		Flowers	34.92 µg/100 g	non-hydrolyzed;		
			69.66 µg/100 g	hydrolyzed		
	Phenolic acid	Leaves	0.4–2.1 mg/100 g	n.s.	Methanolic extraction; HPLC-DAD	[13]
Ferulic acid	Phenolic acid	Leaves	58.27 ± 2.98 µg/g DW 49.82 ± 3.55 µg/g DW	Free Bound	80% boiling aqueous MeOH + EtOAc; acid hydrolysis; HPLC-DAD	[85]
	Phenolic acid	Bulbs	62.62 ± 4.69 µg/g DW 30.68 ± 2.34 µg/g DW	Free Bound	80% boiling aqueous MeOH + EtOAc; acid hydrolysis; HPLC-DAD	[85]
	Phenolic acid	Leaves Flowers	40.16 µg/100 g 163.38 µg/100 g 38.89 µg/100 g 52.97 µg/100 g	non-hydrolyzed; hydrolyzed. non-hydrolyzed; hydrolyzed	70% ethanol, repercolation; HPLC-UV-MS; acid hydrolysis	[89]
	Phenolic acid	Leaves	8.3–47.8 mg/100 g	n.s.	Methanolic extraction; HPLC-DAD	[13]
Vanillic acid	Phenolic acid	Leaves	61.48 ± 6.78 µg/g DW 60.05 ± 7.02 µg/g DW	Free Bound	80% boiling aqueous MeOH + EtOAc; acid hydrolysis; HPLC-DAD	[85]
	Phenolic acid	Bulbs	87.93 ± 8.46 µg/g DW trace	Free Bound	80% boiling aqueous MeOH + EtOAc; acid hydrolysis; HPLC-DAD	[85]
	Phenolic acid	Leaves	0.9–13.5 mg/100 g	n.s.	Methanolic extraction; HPLC-DAD	[13]
*p*-Hydroxybenzoic acid	Phenolic acid	Bulbs	30.36 ± 2.91 µg/g DW	free	MeOH/EtOAc; HPLC	[85]
Catechin hydrate	Flavanol	Leaves	0.1–23.6 mg/100 g	n.s.	Methanolic extraction; HPLC-DAD	[13]
Epicatechin	Flavanol	Leaves	2.4–12.3 mg/100 g	n.s.	Methanolic extraction; HPLC-DAD	[13]
Rutin	Flavonol glycoside	Leaves	2.3–29.8 mg/100 g	n.s.	Methanolic extraction; HPLC-DAD	[13]
Kaempferol	Flavonol aglycone	Leaves Flowers	252.76 µg/100 g; 1839.33 µg/100 g;	Hydrolyzed	70% ethanol + acid hydrolysis; HPLC-UV-MS	[89]
Kaempferol derivatives, total	Flavonols	Green leaves Yellow leaves	1856.31 mg/100 g DW 2362.96 mg/100 g DW	n.s.	MeOH + 1% acetic acid, sonication; UPLC-PDA-ESI-MS/MS	[86]
Kaempferol derivatives, total	Flavonols	Stalks Seeds	206.07 mg/100 g DW 73.14 mg/100 g DW	n.s.	MeOH + 1% acetic acid, sonication; UPLC-PDA-ESI-MS/MS	[86]
4′-O-acetyl-kaempferol-3-O-rhamnosyl-glucoside-7-O-trans-p-coumaroyl-glucoside	Acylated flavonol	Green leaves	344.77 ± 6.78 mg/100 g DW	n.s.	MeOH + 1% acetic acid, sonication; UPLC-PDA-ESI-MS/MS	[86]
		yellow leaves	630.44 ± 5.98 mg/100 g DW			
		stalks	13.20 ± 1.87 mg/100 g DW			
		seeds	4.85 ± 0.42 mg/100 g DW			
Sum of chromatographically quantified phenolic compounds	Quantified phenolic profile dominated by kaempferol derivatives	Leaves Stems Flowers Roots	1195.9–1477 mg/100 g DW 31.6–109.7 mg/100 g DW 269.5–917.9 mg/100 g DW 6.5–10.7 mg/100 g DW	n.s.	50% methanol, sonication; UPLC-PDA-Q/TOF-MS	[42]

Notes: DW, dry weight; FW, fresh weight; FAE-ferulic acid equivalents; GAE, gallic acid equivalents; TPC, total phenolic content; HPLC-DAD, high-performance liquid chromatography with diode-array detection; UPLC-PDA-Q/TOF-MS, ultra-performance liquid chromatography with photodiode-array quadrupole/time-of-flight mass spectrometry. Values are reported as in the original studies and should not be directly compared across studies because of differences in plant organ, developmental stage, extraction solvent, analytical methodology, and reporting basis. Folin-Ciocalteu values express overall reducing capacity and are reported as either FAE or GAE, according to the reference standard used in the original study; they are not equivalent to the sum of individual phenolic compounds; MeOH, methanol; EtOAc, ethyl acetate; n.s., phenolic fraction not specified in the original study; HPLC-UV-MS, high-performance liquid chromatography with ultraviolet and mass spectrometric detection; UPLC-PDA-ESI-MS/MS, ultra-performance liquid chromatography with photodiode-array detection and electrospray ionization tandem mass spectrometry. Values are reported as in the original studies and should not be directly compared across studies because of differences in plant material, extraction and hydrolysis procedures, analytical methodology, and reporting basis. “Free” and “bound”, as well as “non-hydrolyzed” and “hydrolyzed”, are retained only where these fractions or treatments were explicitly distinguished in the original studies.

**Table 8:** Major pigments, including carotenoids and chlorophylls, identified in *Allium ursinum* L.

Compound	Plant/Part	Condition/Stage	Quantity	Unit	Method	Reference
Total carotenoids	Leaves	March June	6550.5 ± 733.7 9142.3 ± 1167.9	mg kg^−1^ DM	UPLC-PDA-MS/MS	[43]
	Stems	March June	521.8 ± 65.0 853.1 ± 64.4	mg kg^−1^ DM	UPLC-PDA-MS/MS	[43]
	Flowers	March June	817.1 ± 108.4 1289.2 ± 148.5	mg kg^−1^ DM	UPLC-PDA-MS/MS	[43]
	Bulbs	March June	5.7 ± 0.9 6.8 ± 1.0	mg kg^−1^ DM	UPLC-PDA-MS/MS	[43]
	Leaves	Flowering	0.48–0.62	mg g^−1^ FW	Acetone extraction; Spectrophotometry	[70]
	Leaves	Fresh	5.29 ± 0.41	mg/100 g FW	80% acetone extraction; Spectrophotometry	[66]
	Leaves	Different soil types	91.17–263.24	μg g^−1^ FW	80% acetone extraction; Spectrophotometry	[71]
Carotene	Leaves	Fresh Frozen Dried	1.58 ± 0.03 2.42 ± 0.08 4.69 ± 0.82	μg mL^−1^ extract	95% EtOH extraction; UV-Vis spectrophotometry	[22]
β-Carotene	Leaves	Frozen, air-dried and freeze-dried;	155–221	mg/100 g DM	Acetone:hexane (4:6); Spectrophotometry	[51]
All-trans-lutein	Leaves	March June	1170.3 ± 0.1 1404.9 ± 0.4	mg kg^−1^ DM	UPLC-MS-QTOF and MS/MS	[43]
Lutein (11 populations)	Leaves Flowers	Flowering	384.3–655.7 25.6–46.4	µg g^−1^ DM	HPLC-PDA-MS	[41]
α-Cryptoxanthin	Leaves	March June	981.5 ± 0.2 1285.7 ± 0.4	mg kg^−1^ DM	UPLC-MS-QTOF and MS/MS	[43]
β-Cryptoxanthin	Leaves	March June	570.4 ± 0.7 593.5 ± 0.1	mg kg^−1^ DM	UPLC-MS-QTOF and MS/MS	[43]
all-trans-β-Carotene	Leaves	March June	2481.0 ± 0.4 4044.5 ± 0.2	mg kg^−1^ DM	UPLC-MS-QTOF and MS/MS	[43]
Total chlorophyll	Leaves	Fresh	174.49 ± 17.09	mg/100 g FW	80% acetone extraction; Spectrophotometry	[66]
	Leaves	Frozen, air-dried and freeze-dried;	638–1532	mg/100 g DM	Acetone:hexane (4:6); Spectrophotometry	[51]
	Leaves	Flowering	1.07–1.42	mg g^−1^ FW	Acetone extraction; Spectrophotometry	[70]
Chlorophyll *a*	Leaves	Different soil types	289.75–642.39	μg g^−1^ FW	80% acetone extraction; Spectrophotometry	[71]
	Leaves	Fresh Frozen Dried	7.74 ± 0.11 3.64 ± 0.15 21.33 ± 0.46	μg mL^−1^ extract	95% EtOH extraction; UV-Vis spectrophotometry	[22]
Chlorophyll *b*	Leaves	Different soil types	358.15–458.57	μg g^−1^ FW	80% acetone extraction; Spectrophotometry	[71]
	Leaves	Fresh Frozen Dried	4.37 ± 0.03 2.66 ± 0.20 14.83 ± 1.66	μg mL^−1^ extract	95% EtOH extraction; UV-Vis spectrophotometry	[22]
Total chlorophyll	Leaves	Fresh Frozen Dried	12.12 ± 0.08 6.29 ± 0.35 36.12 ± 1.19	μg mL^−1^ extract	95% EtOH extraction; UV-Vis spectrophotometry	[22]

Notes: DM, dry matter; FW, fresh weight. HPLC-PDA-MS, High-Performance Liquid Chromatography coupled with Photodiode Array Detection and Mass Spectrometry; MS/MS, Tandem Mass Spectrometry; UPLC-MS-QTOF, Ultra-Performance Liquid Chromatography coupled to Quadrupole Time-of-Flight Mass Spectrometry. Values reported for March and June by Lachowicz et al. correspond to two distinct collection times and are presented in chronological order. Lutein values reported by Radušienė et al. represent minimum-maximum ranges recorded across 11 Lithuanian populations for leaves and 10 flowering populations for flowers. Values expressed per milliliter of extract are method-dependent and should not be directly compared with concentrations expressed per unit of fresh or dry plant material.

**Table 9:** Other bioactive compounds reported in *Allium ursinum* L.

Class	Compound	Plant Organ	Condition/Stage	Quantity	Method	References
Lectin	AUAI (heterodimeric mannose-specific lectin)	Bulbs	Resting and developing stage	nq	Mannose-Sepharose affinity chromatography, SDS-PAGE, N-terminal sequencing and cDNA analysis	[92,93,94]
	AUAII (homodimeric mannose-specific lectin)	Bulbs	Resting and developing stage	nq	Mannose-Sepharose affinity chromatography, SDS-PAGE, N-terminal sequencing and cDNA analysis	[92,93,94]
	AUAL (homodimeric mannose-specific lectin)	Leaves	Collected in April	nq	Mannose-Sepharose affinity chromatography, SDS-PAGE and molecular cloning	[95]
Protein complex	AUAI-alliinase complex	Bulbs	Formed in vitro after tissue homogenization	nq	Mannose-Sepharose affinity chromatography, SDS-PAGE and molecular cloning	[93]
Steroidal sapogenin	Diosgenin	Bulbs	March-October; different phenological stages	0.0018–0.00796% (fresh material); 0.039–0.137% (methanolic extracts)	Acid hydrolysis + TLC densitometry (540 nm)	[96]
Steroidal sapogenin	Diosgenin	Leaves	March-May; different phenological stages	ND to 0.00029% (fresh material); ND to 0.002% (methanolic extracts)	Acid hydrolysis + TLC densitometry (540 nm)	[96]
Triterpenoids	Betulinic, oleanolic and ursolic acids	Leaves; Stems; Flowers; Bulbs	March June	Total triterpenoids: 2988.9–5330.2; 2688.0–3312.6; 1814.1–2328.3 and 636.1–865.2 mg kg^−1^ DM, respectively	Ethyl acetate/hexane extraction; UPLC-PDA-QTOF-MS	[43]
Galactolipid	1,2-di-O-α-linolenoyl-3-O-β-D-galactopyranosyl-sn-glycerol (DLGG)	Leaves	Fresh leaves (March)	2.23 g isolated; approximately 1.26% of the dry ethanolic extract	Ethanolic extraction, liquid-liquid partition, column chromatography, ESI-MS, NMR	[25]
Phytosterol	β-Sitosterol-3-O-β-D-glucopyranoside	Leaves	Fresh leaves (March)	17 mg isolated	Ethanolic extraction, liquid-liquid partition, column chromatography, ESI-MS, NMR	[25]
Amino acids	Fourteen amino acids, including phenylalanine	Bulbs; Leaves	Vegetative stage before flower stalk development (spring 2021); eight Croatian populations	Phenylalanine: 19.2 ± 2.7 and 8.30 ± 0.93 µmol/100 g DW, respectively; dominant amino acids: arginine in bulbs and glutamic acid in leaves	UPLC-FLD after pre-column derivatization	[84]
Organic acids	Glycolic, oxalic, malonic, benzoic, succinic, glyceric, malic, threonic, 2-oxoglutaric, shikimic, citric and galacturonic acids	Leaves	Dried overnight at 50 °C; hot-water extract prepared at 70 °C	Tentatively identified; relative GC-MS peak areas only	GC-MS after derivatization	[73]

Notes: nq, not quantified; SDS-PAGE, sodium dodecyl sulfate-polyacrylamide gel electrophoresis; cDNA, complementary DNA; ESI-MS, electrospray ionization mass spectrometry; NMR, nuclear magnetic resonance; UPLC-FLD, ultra-performance liquid chromatography with fluorescence detection; DLGG, 1,2-di-O-α-linolenoyl-3-O-β-D-galactopyranosyl-sn-glycerol. Diosgenin values reported by Sobolewska et al. were obtained after acid hydrolysis of methanolic extracts and therefore represent released sapogenin rather than intact steroidal glycosides. Values reported for DLGG and β-sitosterol-3-O-β-D-glucopyranoside represent isolation yields and/or relative abundance in extracts rather than direct tissue concentrations. Lectin studies provide structural characterization but no concentrations expressed on a fresh- or dry-weight basis; moreover, the occurrence of AUAI-alliinase complexes in vivo was not confirmed because these complexes may form immediately after tissue homogenization. Amino-acid values are expressed as µmol/100 g DW and represent mean ± SE (*n* = 3) across eight Croatian populations. Organic acids reported by Hangweirer et al. were tentatively identified by GC-MS spectral and retention-index matching; the corresponding peak areas represent relative analytical signals rather than absolute concentrations in the extract or plant tissue. Accordingly, direct quantitative comparisons among compound classes should be interpreted with caution.

**Table 10:** Chemical antioxidant capacity of different organs and extracts of *A*. *ursinum* L. measured by cell-free assays.

Plant Organ	Condition/Extraction	DPPH	FRAP	ABTS	ORAC	Reference
Leaves	Lyophilized material; mean of 8 populations	5.0 µmol TE g^−1^ DW	8.8 µmol TE g^−1^ DW	-	293 nmol TE g^−1^ DW	[84]
	Fresh	260 µmol TE g^−1^ FW	0.5 mmol Fe^2+^ g^−1^ FW	-	-	[22]
	Frozen	280 µmol TE g^−1^ DW	1.0 mmol Fe^2+^ g^−1^ DW	-	-	[22]
	Dried	700 µmol TE g^−1^ DW	2.60 mmol Fe^2+^ g^−1^ DW	-	-	[22]
	Pressurized-liquid extract	0.25 mM TE g^−1^ FW	2.02 mM TE g^−1^ FW	2.65 mM TE g^−1^ FW	60.35 mM TE g^−1^ FW	[103]
	80% methanolic extract of dried leaves	66.66–77.21 µg mL^−1^ (EC_50_)	-	-	-	[104]
	70% ethanolic extract of dried leaves	46.10–77.21 µg mL^−1^ (EC_50_)	-	-	-	[104]
	Traditional tincture from fresh leaves	44.99–50.00 µg mL^−1^ (EC_50_)	-	-	-	[104]
	Fresh leaves; 80% methanolic extract	17.39 mg mL^−1^ (EC_50_)	-	-	-	[66]
	Aqueous extract	154.25 µg mL^−1^ (IC_50_)	-	-	-	[105]
	Methanol extract	111.04 µg mL^−1^ (IC_50_)	-	-	-	[105]
	Chloroform extract	391.79 µg mL^−1^ (IC_50_)	-	-	-	[105]
	Methanolic extract, April	10.7 ± 1.57% inhibition	-	-	-	[87]
	Methanolic extract, May	15.05 ± 1.21% inhibition	-	-	-	[87]
	Methanolic extract, June	17.3 ± 0.85% inhibition	-	-	-	[87]
	Freeze-dried material; mean of March and June harvests	-	11.7 µmol g^−1^ DM	91.7 µmol g^−1^ DM	-	[43]
	Air-dried, mean of 4 locations	116 µmol TE g DM	1333 µmol TE g^−1^ DM	1905 µmol TE g^−1^ DM	-	[51]
	Freeze-dried, mean of 4 locations	43 µmol TE g^−1^ DM	1195 µmol TE g^−1^ DM	1580 µmol TE g^−1^ DM	-	[51]
	Natural populations, before flowering/flowering	-	-	up to 2230.66 µmol TE L^−1^	-	[70]
	Natural populations, 11 populations	-	-	718.6 µmol TE g^−1^ DM	-	[41]
Bulb	Lyophilized material; mean of 8 populations	9.80 µmol TE g^−1^ DW	11.8 µmol TE g^−1^ DW	-	87.2 nmol TE g^−1^ DW	[84]
	Freeze-dried material; mean of March and June harvests	-	5.8 µmol TE g^−1^ DM	50.9 µmol TE g^−1^ DM	-	[43]
Stems	Freeze-dried material; mean of March and June harvests	-	2.7 µmol Fe^2+^ g^−1^ FW	37.1 µmol TE g^−1^ FW	-	[43]
Flowers	Freeze-dried material; mean of March and June harvests	-	5.3 µmol Fe^2+^ g^−1^ FW	45.1 µmol TE g^−1^ FW	-	[43]
	Methanolic extract, May	25.9 ± 1.06% inhibition	-	-	-	[87]
	Natural populations, 10 flowering populations	-	-	473.0 µmol TE g^−1^ DM	-	[41]
Whole plant	Raw, natural populations (5 locations)	-	0.129–0.153 mmol Fe^2+^ g^−1^ DW	-	-	[10]

Notes: DPPH, 2,2-diphenyl-1-picrylhydrazyl radical scavenging assay; FRAP, ferric reducing antioxidant power assay; ABTS, 2,2′-azinobis (3-ethylbenzothiazoline-6-sulfonic acid) radical scavenging assay; ORAC, oxygen radical absorbance capacity assay; TE, Trolox equivalents; FW, fresh weight; DW, dry weight; DM, dry matter; IC_50_, concentration required to inhibit 50% of radical activity; EC_50_, effective concentration providing 50% antioxidant activity. These cell-free assays characterize reaction-specific chemical antioxidant capacity and should not be interpreted as direct evidence of biological antioxidant efficacy. Antioxidant activity values are reported in the original units used by the respective studies. Because different analytical assays, extraction procedures, expression units, and sample preparation protocols were employed, direct quantitative comparisons among studies should be interpreted with caution. Variability in antioxidant activity is further influenced by plant organ, extraction solvent, phenological stage, geographical origin, processing treatment, and analytical methodology. Higher antioxidant values in dried samples should be interpreted with caution, as water loss may contribute to apparent concentration effects when results are expressed on an FW basis, but cannot explain differences among values already normalized to DW or DM.

**Table 11:** Antimicrobial and antifungal activities of *Allium ursinum* extracts and plant materials.

Extract/Material	Microorganism	Reported Antimicrobial Outcome	Method	Reference
**A. Broth dilution MIC/MBC/MFC assays**				
Aqueous extract	*Candida albicans*	MIC = 0.5–4.0 mg/mL, planktonic cells; MIC = 1.0 > 4.0 mg/mL, adherent cells	MIC assay	[108]
Aqueous extract, high-pressure extraction	*Listeria monocytogenes*	MIC = 28 mg/mL; MBC = 29 mg/mL	Broth macrodilution	[109]
Aqueous extract, high-pressure extraction	*Escherichia coli*, *Salmonella* spp., *Enterococcus* spp., *Proteus* spp.	MIC ≈ 29 mg/mL; MBC ≈ 30 mg/mL	Broth macrodilution	[109]
Hydroalcoholic leaf extract	*E*. *coli* ATCC 25922	MIC = 6.25%; MBC = 12.5%	Microdilution	[110]
Hydroalcoholic leaf extract	*Staphylococcus aureus* ATCC 25923	MIC = 25%; MBC = 50%	Microdilution	[110]
Hydroalcoholic leaf extract	*Candida albicans* ATCC 10231	MIC = 6.25%; MBC = 12.5%	Microdilution	[110]
Hydroalcoholic leaf extract	*Candida parapsilosis* ATCC 22019	MIC = 6.25%; MBC = 25%	Microdilution	[110]
96% ethanol leaf extract	*Salmonella enteritidis* ATCC 13076	MIC= 1.56 mg/mL MBC = 3.13 mg/mL	Microdilution	[111]
96% ethanol leaf extract	*E*. *coli*, *Proteus mirabilis*, *Enterococcus faecalis*	MIC = 1.56 mg/mL; MBC = 6.25 mg/mL	Microdilution	[111]
96% ethanol leaf extract	*Candida albicans* ATCC 10031	MIC= 6.25 mg/mL MFC = 12.50 mg/mL	Microdilution	[111]
**B. Agar-based diffusion and growth-inhibition assays**				
Hydroalcoholic leaf extract	*Candida* spp.	Inhibition zones = 20–32 mm	Disk diffusion	[110]
Hydroalcoholic leaf extract	*Staphylococcus aureus*	Inhibition zones = 15–22 mm	Disk diffusion	[110]
50% ethanol flower extract	*Aspergillus niger*	MIC = 100 µL/mL	Agar-dilution assay	[112]
50% ethanol flower extract	*Botrytis cinerea*	MIC = 60 µL/mL	Agar-dilution assay	[112]
50% ethanol flower extract	*Botrytis paeoniae*	MIC = 70 µL/mL	Agar-dilution assay	[112]
50% ethanol flower extract	*Fusarium oxysporum* f. sp. *tulipae*	MIC = 140 µL/mL	Agar-dilution assay	[112]
50% ethanol flower extract	*Penicillium gladioli*	MIC = 90 µL/mL	Agar-dilution assay	[112]
50% ethanol flower extract	*Sclerotinia sclerotiorum*	MIC = 60 µL/mL	Agar-dilution assay	[112]
5% ethanolic leaf, bulb and root extracts	*Candida albicans*, *E*. *coli*, *S*. *aureus*, *Streptococcus pyogenes*, *Bacillus subtilis*	Inhibition ranged from slight (0–9 mm) to moderate (10–15 mm) and large (>16 mm), depending on plant organ and microorganism	Disk diffusion	[113]
Acetone extract (flowers)	*Staphylococcus aureus*	Inhibition zone = 19 ± 1.2 mm; MIC = 625 µg/mL	Modified diffusion assay	[18]
Acetone extract (leaves)	*Staphylococcus aureus*	Inhibition zone = 19 ± 1.2 mm; MIC = 625 µg/mL	Modified diffusion assay	[18]
Chloroform extract (leaves)	*Candida albicans*	No inhibition zone reported; MIC = 312 µg/mL	Agar cup method	[18]
Chloroform extract (flowers)	*Candida albicans*	No inhibition zone reported; MIC > 625 µg/mL	Agar cup method	[18]
**C. Food-matrix studies**				
Ramsons powder in rabbit meat burgers	Enterobacteriaceae, *Pseudomonas* spp., lactic acid bacteria, total aerobic psychrotrophic bacteria	No significant reduction in microbial counts; bacterial counts increased after 7 days of storage	Food system evaluation	[114]
Fresh bulbs incorporated into mayonnaise	Aerobic mesophilic microorganisms at 30 °C	Reduced microbial counts compared with control during storage	Food matrix microbiological evaluation	[115]

Notes: MIC, minimum inhibitory concentration; MBC, minimum bactericidal concentration; MFC, minimum fungicidal concentration. MIC, MBC and MFC values are reported in the original units used by the respective studies (mg/mL, %, or µL/mL). Inhibition zones are expressed as millimeters (mm). Results obtained by broth microdilution, agar dilution and disk diffusion assays are not directly comparable because they are based on different experimental principles. Food-system studies evaluate antimicrobial performance under complex food matrix conditions and therefore should not be directly compared with in vitro susceptibility assays.

**Table 12:** Effects of plant parts and extracts of *Allium ursinum* L. on the cardiovascular system.

Plant Material/Extract	Study Model	Experimental Conditions	Main Findings	Reference
Dried leaf powder	Rat isolated heart (ischemia/reperfusion; Langendorff preparation)	2% in diet, 8 weeks	↓ ventricular fibrillation; ↓ ischemic area; delayed arrhythmias onset;	[116]
Dietary wild garlic	Spontaneously hypertensive rats	1% *w*/*w* in diet, 45 days	↓ systolic blood pressure; ↓ total cholesterol; ↑ HDL	[117]
Alcoholic leaf extract	Human platelet aggregation	In vitro, 5 mg/mL	↓ ADP-induced aggregation; ↓ ARA and epinephrine-induced aggregation to a lesser extent	[24]
Active fractions from ethanolic leaf extract	Human platelet aggregation	In vitro; Bioassay-guided isolation	Galactolipid and phytosterol identified as inhibitors of ADP-induced platelet aggregation	[25]
Wild garlic leaf lyophilizate (WGLL)	Hypercholesterolemic rabbits	2% WGLL + 2% cholesterol in diet, 8 weeks	↓ ApoB, LDL and atherosclerotic plaque area; ↑ cardiac function; ↑ HO−1	[118]
Wild garlic leaf lyophilizate (WGLL)	Rat pulmonary arterial hypertension	WGLL-enriched diet, 8 weeks	↑ right ventricular function; ↓ pulmonary vascular remodeling; effects comparable to sildenafil	[119]
Water and methanolic leaf extracts	H9c2 cardiomyoblasts (doxorubicin toxicity)	4 h pre-treatment, 50 μg/mL	Water extract: ↓ intracellular and mitochondrial ROS; methanolic extract: no ROS reduction	[31]
Methanolic extract of aerial parts	Rat ischemia/reperfusion injury	125–500 mg/kg, 28 days	↑ cardiac contractility; ↑ coronary flow; ↓ oxidative stress	[20]

Notes: ADP, adenosine diphosphate; ApoB, apolipoprotein B; HDL, high-density lipoprotein cholesterol; HO-1, heme oxygenase-1; LDL, low-density lipoprotein cholesterol; ROS, reactive oxygen species; ↑ indicates an increase, whereas ↓ indicates a decrease.

**Table 13:** Cytoprotective, antioxidant, and redox-modulating effects of *A*. *ursinum* L.

Plant Material/Extract	Experimental Model	Treatment	Main Findings	Reference
Phenolic ethanolic leaf extract	Human erythrocytes exposed to terbuthylazine	Terbuthylazine 4.25 mg/L; co-treatment with extract for 1 h	↓ MDA from 59.69 to 43.45 nmol/g Hb; ↓ Hb from 165.08 to 128.64 g/L ↑ CAT	[26]
Aqueous leaf extract	H9c2 cardiomyoblasts exposed to doxorubicin	50 μg/mL; 4 h pretreatment	↓ intracellular ROS; ↓ mitochondrial ROS; no improvement in cell viability	[31]
Methanolic leaf extract	H9c2 cardiomyoblasts exposed to doxorubicin	50 μg/mL; 4 h pretreatment	No reduction in intracellular or mitochondrial ROS; no protection of cell viability	[31]
Wild garlic leaf lyophilizate (WGLL)	Hypercholesterolemic rabbits	2% WGLL + 2% cholesterol in diet; 8 weeks	↑ HO-1; ↓ SOD-1 expression toward control levels; ↓ LDL; ↓ ApoB; ↓ aortic plaque area; improved cardiac function	[118]
Methanolic extract of aerial parts	Rat myocardial ischemia/reperfusion injury	125–500 mg/kg; 28 days	↓ TBARS and pro-oxidative markers; ↑ CAT and SOD; improved post-ischemic recovery	[20]

Notes: MDA, malondialdehyde; Hb, hemoglobin; CAT, catalase; ROS, reactive oxygen species; HO-1, heme oxygenase-1; SOD, superoxide dismutase; LDL, low-density lipoprotein cholesterol; ApoB, apolipoprotein B; TBARS, thiobarbituric acid-reactive substances; WGLL, wild garlic leaf lyophilizate; ↑ indicates an increase, whereas ↓ indicates a decrease. The table summarizes studies relevant to cytoprotective or redox-modulating effects. Cardiovascular studies are included only when they provide mechanistic evidence relevant to redox modulation and should not be interpreted as direct evidence of anti-inflammatory activity.

**Table 14:** Safety considerations for *Allium ursinum* L. and toxicological risks associated with its misidentification.

Species	Hazard	Toxic Constituent	Toxicological Effect	Prevention	Reference
Correctly identified *A*. *ursinum*	Generally recognized as safe; potential adverse reactions should be considered	Diallyl tri- and tetrasulfides; potential allergenic constituents	No human hemolysis reported; potential allergic reactions and potential enhancement of anticoagulant therapy.	Caution in susceptible individuals and during anticoagulant therapy	[16]
*Convallaria majalis*	Accidental substitution with wild garlic leaves, especially before flowering	Cardiac glycosides, mainly convallatoxin, convalloside and lokunjoside; saponins	Digitalis-like toxicity; gastrointestinal symptoms; bradycardia/irregular heartbeat; arrhythmias; cardiovascular toxicity	Morphological and histological authentication; avoid harvesting unidentified young leaves	[32]
*Colchicum autumnale*	Accidental substitution with wild garlic leaves	Colchicine	Gastrointestinal symptoms within 4–12 h; renal failure, cardiovascular collapse, respiratory insufficiency, multiorgan dysfunction; potentially fatal	Confirmation of suspected poisoning by LC-HRMS/MS or LC-ITMS^n^ when poisoning is suspected	[30]
*Arum maculatum*	Potential adulteration/misidentified plant in edible wild garlic material	Oxalates and saponins reported particularly in the fruits	Allergic reactions possible after consumption	Metabolomic authentication; isovitexin as an adulteration marker	[121]

**Table 15:** Authentication approaches for distinguishing *Allium ursinum* L. from poisonous look-alike species.

Authentication Approach	Diagnostic Criterion	Application	Reference
Macroscopic botanical identification	Characteristic garlic-like odor; smooth, flat, elliptic-lanceolate leaves gradually narrowed into the petiole	Preliminary field identification during harvesting	[16]
Histological and microscopic analysis	Hypostomatic leaves in *A*. *ursinum*; *C*. *majalis* and *C*. *autumnale* amphistomatic; characteristic wavy abaxial epidermal cell walls in *A*. *ursinum*.	Authentication of fresh, dried or fragmented plant material	[32]
Untargeted metabolomic fingerprinting (^1^H NMR, LC-MS)	Isovitexin, vicenin II, azetidine-2-carboxylic acid and trigonelline as biomarkers of *Convallaria majalis*; isovitexin as biomarker of *Arum maculatum* adulteration	Detection of adulteration and food authenticity control	[121]

**Table 16:** Documented food applications and investigated formulation strategies for *Allium ursinum* L.

Food Matrix and Product	Form of *A*. *ursinum*	Processing/Conditions	Functional Effects	Quality, Safety and Sensory Impact	Reference
Extruded corn snacks	Wild garlic leaf powder (1–4%)	Extrusion; simulated gastric and duodenal digestion	Before digestion: ↑ polyphenols, ↑ phenolic acids, ↑ antioxidant activity After simulated digestion: ↓ polyphenols, ↓ free phenolic acids, ↓ antiradical activity	Acceptable physicochemical properties	[29]
Soft rennet-curd cheese	Dried leaves	Cheese manufacture and storage	↑ antioxidant capacity (DPPH assay), enhanced proteolysis	No adverse effect on microbiological quality	[126]
Soft cow cheese	Dried leaves	Cheese manufacture and storage	Enriched volatile profile	Modified color and texture; maintained sensory quality	[28]
Sheep milk cheese	Leaves	Cheese manufacture and storage	Modified volatile profile	Stronger herbal aroma and piquant taste; maintained sensory quality	[60]
Reformulated meat product	Leaves	Meat processing	Improved oxidative stability; increased phenolic compounds	Improved water-binding capacity; shelf-life extension potential	[127]
Minced meat semi-finished products	Leaves	Meat processing	↑ nutritional value; source of vitamins, fiber and bioactive compounds	Extended shelf life; acceptable organoleptic properties	[128]
Mayonnaise	Fresh bulbs	Emulsification and storage	High antioxidant activity; antimicrobial effect	Improved physicochemical and microbiological quality	[115]
Functional pasta	Powder, extract, encapsulated extract	Cooking	↑ phenolics, ↑ flavonoids, ↑ antioxidant activity	Powder gave best sensory acceptance	[27]
Egg pasta	Fresh leaves	Cooking	↑ polyphenols, ↑ antioxidant capacity	Improved overall sensory quality	[59]
Fortified pasta	Powder, blanched leaves, blanching water	Cooking	↑ total phenolic content ↑ total flavonoid content, ↑ antioxidant activity	Increased cooking loss; color changes; good sensory acceptance	[58]
*A*. *ursinum* honey	Natural botanical transfer of floral metabolites	HS-SPME and USE-GC/MS	Matrix-specific phytochemical profile	Potential botanical-origin markers; relative signals only.	[129]
Liquid extract	SWE extract	Subcritical water extraction	High recovery of phenolics, flavonoids and antioxidant compounds	Potential ingredient for functional foods	[130]
Encapsulated extract	Extract	Spray congealing	Improved solubility (>18-fold), stability and antimicrobial activity	Better handling and storage stability	[131]
Encapsulated powder	SWE extract + maltodextrin	Spray drying	↑ retention of phenolics and flavonoids; preserved antioxidant activity	Excellent storage stability	[125]
Selenium-enriched functional food	Leaves	Selenium biofortification	↑ selenium content, ↑ antioxidant status, ↑ ascorbic acid	Functional food potential	[132]

Notes: DPPH, 2,2-diphenyl-1-picrylhydrazyl radical-scavenging assay; HS-SPME, headspace solid-phase microextraction; USE, ultrasound-assisted extraction; GC/MS, gas chromatography-mass spectrometry; SWE, subcritical water extraction; ↑ indicates an increase, whereas ↓ indicates a decrease.

## Data Availability

No new data were created or analyzed in this study. Data sharing is not applicable to this article.

## Abbreviations

The following abbreviations are used in this manuscript:

ACSO	S-alk(en)yl-L-cysteine sulfoxide
ABTS	2,2′-azinobis(3-ethylbenzothiazoline-6-sulfonic acid) radical scavenging assay
ACE	Angiotensin-converting enzyme
ADP	Adenosine diphosphate
ApoB	Apolipoprotein B
CAT	Catalase
DADS	Diallyl disulfide
DATS	Diallyl trisulfide
DM	Dry matter
DPPH	2,2-diphenyl-1-picrylhydrazyl radical scavenging assay
DW	Dry weight
EC_50_	Effective concentration providing 50% antioxidant activity
FRAP	Ferric reducing antioxidant power assay
FW	Fresh weight
GAE	Gallic acid equivalents
GBIF	Global Biodiversity Information Facility
GC-FID	Gas chromatography-flame ionization detection
GC-MS	Gas chromatography-mass spectrometry
Hb	Hemoglobin
HDL	High-density lipoprotein cholesterol
HO-1	Heme oxygenase-1
HPLC	High-performance liquid chromatography
HPLC-DAD	High-performance liquid chromatography with diode-array detection
IC_50_	Concentration required to inhibit 50% of radical activity
ICP-AES	Inductively coupled plasma atomic emission spectroscopy
ICP-MS	Inductively coupled plasma mass spectrometry
ICP-OES	Inductively coupled plasma optical emission spectroscopy
I/R	Ischemia/reperfusion
LDL	Low-density lipoprotein cholesterol
MBC	Minimum bactericidal concentration
MDA	Malondialdehyde
MFC	Minimum fungicidal concentration
MIC	Minimum inhibitory concentration
MS	Mass spectrometry
ND	Not detected
NMR	Nuclear magnetic resonance
ORAC	Oxygen radical absorbance capacity assay
ROS	Reactive oxygen species
RSA	Radical scavenging activity
SDS-PAGE	Sodium dodecyl sulfate-polyacrylamide gel electrophoresis
SOD	Superoxide dismutase
SWE	Subcritical water extraction
TBARS	Thiobarbituric acid-reactive substances
TE	Trolox equivalents
TEAC	Trolox equivalent antioxidant capacity
TFC	Total flavonoid content
TPC	Total phenolic content
UPLC-PDA-ESI-MS/MS	Ultra-performance liquid chromatography with photodiode-array detection and electrospray ionization tandem mass spectrometry
UPLC-PDA-Q/TOF-MS	Ultra-performance liquid chromatography with photodiode-array detection and quadrupole/time-of-flight mass spectrometry
VOSviewer	Software used for bibliometric network visualization
